# Synergistic toxicity with copper contributes to NAT2-associated isoniazid toxicity

**DOI:** 10.1038/s12276-024-01172-8

**Published:** 2024-03-01

**Authors:** Jihoon G. Yoon, Dong Geon Jang, Sung-Gyu Cho, Chaeyoung Lee, Shin Hye Noh, Soo Kyung Seo, Jung Woo Yu, Hyeon Woo Chung, KyeoRe Han, Soon Sung Kwon, Dai Hoon Han, Jaeseong Oh, In-Jin Jang, Sang-Hoon Kim, Young-Koo Jee, Hyun Lee, Dong Won Park, Jang Won Sohn, Ho Joo Yoon, Chul Hoon Kim, Jae Myun Lee, Sang-Heon Kim, Min Goo Lee

**Affiliations:** 1 Department of Pharmacology, BK21 Project of Yonsei Advanced Medical Science, Woo Choo Lee Institute for Precision Drug Development, Yonsei University College of Medicine, Seoul, Republic of Korea; 2 Department of Genomic Medicine, Seoul National University Hospital, Seoul, Republic of Korea; 3 Department of Microbiology and Immunology, Yonsei University College of Medicine, Seoul, Republic of Korea; 4 Severance Biomedical Science Institute, Yonsei University College of Medicine, Seoul, Republic of Korea; 5 Department of Laboratory Medicine, Yonsei University College of Medicine, Seoul, Republic of Korea; 6 Division of Hepatobiliary and Pancreatic Surgery, Department of Surgery, Yonsei University College of Medicine, Seoul, Korea; 7 Department of Clinical Pharmacology and Therapeutics, Seoul National University College of Medicine and Hospital, Seoul, Republic of Korea; 8 Department of Internal Medicine, Eulji University School of Medicine, Seoul, Republic of Korea; 9 Department of Internal Medicine, Dankook University College of Medicine, Cheonan, Republic of Korea; 10 Department of Internal Medicine, Hanyang University College of Medicine, Seoul, Republic of Korea

**Keywords:** Genetics research, Mechanisms of disease, Predictive markers, Tuberculosis

## Abstract

Anti-tuberculosis (AT) medications, including isoniazid (INH), can cause drug-induced liver injury (DILI), but the underlying mechanism remains unclear. In this study, we aimed to identify genetic factors that may increase the susceptibility of individuals to AT-DILI and to examine genetic interactions that may lead to isoniazid (INH)-induced hepatotoxicity. We performed a targeted sequencing analysis of 380 pharmacogenes in a discovery cohort of 112 patients (35 AT-DILI patients and 77 controls) receiving AT treatment for active tuberculosis. Pharmacogenome-wide association analysis was also conducted using 1048 population controls (Korea1K). *NAT2* and *ATP7B* genotypes were analyzed in a replication cohort of 165 patients (37 AT-DILI patients and 128 controls) to validate the effects of both risk genotypes. *NAT2* ultraslow acetylators (UAs) were found to have a greater risk of AT-DILI than other genotypes (odds ratio [OR] 5.6 [95% confidence interval; 2.5–13.2], *P* = 7.2 × 10^−6^). The presence of *ATP7B* gene 832R/R homozygosity (rs1061472) was found to co-occur with *NAT2* UA in AT-DILI patients (*P* = 0.017) and to amplify the risk in *NAT2* UA (OR 32.5 [4.5–1423], *P* = 7.5 × 10^−6^). In vitro experiments using human liver-derived cell lines (HepG2 and SNU387 cells) revealed toxic synergism between INH and Cu, which were strongly augmented in cells with defective NAT2 and ATP7B activity, leading to increased mitochondrial reactive oxygen species generation, mitochondrial dysfunction, DNA damage, and apoptosis. These findings link the co-occurrence of *ATP7B* and *NAT2* genotypes to the risk of INH-induced hepatotoxicity, providing novel mechanistic insight into individual AT-DILI susceptibility.

**Figure:**
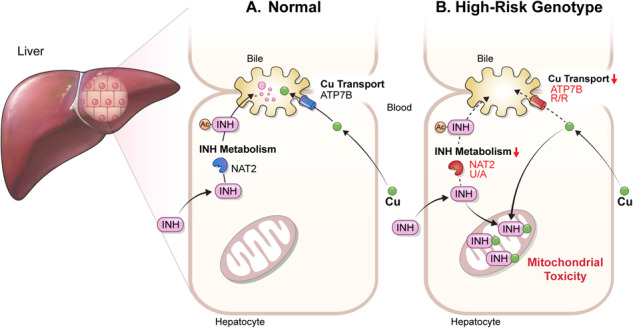
Yoon et al. showed that individuals who carry *NAT2* UAs and *ATP7B* 832R/R genotypes are at increased risk of developing isoniazid hepatotoxicity, primarily due to the increased synergistic toxicity between isoniazid and copper, which exacerbates mitochondrial dysfunction-related apoptosis.

Yoon et al. showed that individuals who carry *NAT2* UAs and *ATP7B* 832R/R genotypes are at increased risk of developing isoniazid hepatotoxicity, primarily due to the increased synergistic toxicity between isoniazid and copper, which exacerbates mitochondrial dysfunction-related apoptosis.

## Genetic factors amplify tuberculosis drug-induced liver injury risk

Tuberculosis is a major health issue, with the bacterium Mycobacterium tuberculosis causing illness and death globally. A key challenge in treating TB is the liver damage caused by drugs used in treatment, particularly isoniazid, which can lead to severe liver injury. While genetic factors like the NAT2 gene have been linked to this drug-induced liver injury, the exact reasons why some people are more susceptible remain unclear. Researchers found that certain genetic variants, particularly in the NAT2 gene and another gene called ATP7B, were more common in patients who suffered liver injury. In conclusion, the research advances our understanding of how genetics can influence the risk of liver injury from TB drugs. This knowledge could lead to more personalized treatment plans and safer TB management in the future. This summary was initially drafted using artificial intelligence, then revised and fact-checked by the author.

## Introduction

Tuberculosis (TB) caused by the infectious bacterium *Mycobacterium tuberculosis* remains a significant global health concern. Indeed, TB continues to be a leading cause of death worldwide, with 1.6 million individuals dying due to TB in 2021^1^. An obstacle to the successful treatment of TB is the occurrence of adverse drug reactions to anti-tuberculosis (AT) medications. Among these reactions, idiosyncratic drug-induced liver injury (DILI) poses a significant challenge, particularly when triggered by the key AT drug isoniazid (INH)^2,3^. INH has been identified as the second most common agent causing DILI, further emphasizing its clinical importance^4^. Notably, ethnicity plays a crucial role, as Asians, particularly people in India, have a greater incidence of DILI^3,5–7^.

The increased risk of developing AT-DILI has been associated with certain genetic factors, such as functionally null variants of the INH-metabolizing enzyme NAT2 (known as ultraslow acetylators, NAT2 UAs)^8^. Meta-analyses and genome-wide association studies (GWASs) have validated this increased risk across diverse ethnic backgrounds^9–11^. However, the *NAT2* genotyping approach lacks specificity, as only a small percentage of individuals with *NAT2* UA genotypes develop AT-DILI. Consequently, the underlying mechanism of AT-DILI remains poorly understood.

Given that AT-DILI can lead to drug discontinuation, acute liver failure, and subsequent high mortality and morbidity, accurately predicting and mitigating risk by considering individual genetic factors is of utmost importance^12,13^. Therefore, the present study aimed to investigate genetic variants and molecular mechanisms that may predispose individuals to a greater susceptibility to AT-DILI.

## Materials and methods

### Patient cohort

Between 2009 and 2015, 35 apparent AT-DILI patients and 77 treatment-tolerant controls were enrolled from multiple medical centers located in the Republic of Korea, including Eulji, Dankook, Hallym, and Hanyang University hospitals. This discovery cohort included 112 patients who were treated with the isoniazid (INH) – rifampin (RIF) – ethambutol (EMB) – pyrazinamide (PZA) regimen for active TB. Between 2016 and 2017, additional patients (*n* = 165) were recruited from the above hospitals; 37 AT-DILI patients and 128 treatment-tolerant controls were included. This replication cohort included 101 patients with active TB and 64 health care workers with latent TB infection; these patients were also included in a previous report (Table 1)^14^. Informed consent was obtained from all participants, and the study was conducted in accordance with relevant ethical guidelines. The study was reviewed by the Institutional Review Board of Hanyang University Hospital (IRB number 2012-01-009/2017-02-018). We excluded patients with other underlying diseases to minimize the effects of clinical variables.

**Table 1:** Clinical characteristics of the patients.

Characteristics	Discovery cohort (*n* = 112)			Replication cohort (*n* = 165)		
	Case	Control	*P* value	Case	Control	*P* value
Number of patients	35	77		37	128	
Sex, Female (%)	19 (54.3%)	34 (44.2%)	0.415	21 (56.8%)	77 (60.2%)	0.709
Age, median (age range, years)	44 (17–84)	45 (18–84)	0.638	46 (16–80)	43 (19–84)	0.277
Diagnosis (Medication)						
Active TB (HREZ, %)	35 (100%)	77 (100%)	>0.999	29 (78.4%)	72 (56.3%)	0.021
Latent TB (INH, %)	0	0		8 (21.6%)	56 (43.7%)	
DILI classification						
Hepatocellular type	33 (94.3%)	NA		29 (78.4%)	NA	0.09
Mixed type	2 (5.7%)	NA		5 (13.5%)	NA	0.435
Cholestatic type	0	NA		3 (8.1%)	NA	0.24
LFT profiles, peak values						
ALT, median (IQR; IU/L)	349 (257–662)	17 (12–26)	<0.001	366 (205–685)	25 (19–35)	<0.001
AST, median (IQR; IU/L)	279 (196–550)	25 (20–29)	<0.001	295 (157–566)	25 (18–34)	<0.001
Total bilirubin, median (IQR; mg/dL)	1.01 (0.59–3.2)	0.5 (0.40–0.80)	<0.001	0.90 (0.25–2.64)	0.64 (0.45–0.80)	<0.001
Investigation method	Targeted sequencing for 380 pharmacogenes			Genotyping for *NAT2*, *ATP7B*		

*DILI* drug-induced liver injury, *TB* tuberculosis, *HREZ*, isoniazid (H) + rifampin (R) + ethambutol (E) + pyrazinamide (Z), *INH* isoniazid, *LFT* liver function test, *ALT* alanine aminotransferase, *AST* aspartate transaminase, *IQR* interquartile range, *NA* not applicable.

### Patient enrollment and evaluation process

A baseline evaluation of TB infection, including medical history, chest X-ray or computed tomography (CT) results, sputum culture, and acid-fast bacillus staining, was performed. The inclusion criteria were as follows: adult patients newly diagnosed with active TB confirmed by a lesion evident via an imaging study (chest X-ray or CT) or positive results from sputum culture or acid-fast bacillus staining. Patients who met any of the following criteria before initiation of AT medication were excluded: (1) abnormal liver function test (LFT) results; (2) chronic liver disease, including fatty liver disease, liver cirrhosis, and alcoholic liver disease; (3) Hepatitis B or C virus carriage; (4) chronic alcoholism; (5) renal dysfunction or chronic kidney disease; (7) other chronic disorders requiring systemic therapy; and (8) poor medication compliance. Latent TB infection was diagnosed via an interferon-gamma releasing assay (QuantiFERON-TB Gold In-Tube tests; Cellestis Ltd., Carnegie, VIC, Australia), as described previously^14^.

Patients with active TB received a first-line standard treatment regimen of the isoniazid (H) + rifampin (R) + ethambutol (E) + pyrazinamide (Z) combination for the initial 2 months, followed by an HR (±E) combination for 4 months or longer; latent TB infection was treated with INH monotherapy for 9 months following WHO guidelines^15^. The administration period and cessation point of AT medication were determined based on the therapeutic response or clinical manifestations assessed by physicians. We followed international and Korean guidelines for TB treatment and the hepatotoxicity of anti-TB medications^16,17^. In brief, we discontinued treatment until the patients had recovered from DILI and then rechallenged them with the medications one by one while closely monitoring their symptoms and liver function tests. When the causative drug was identified during rechallenge, we removed it from the regimen and treated the patients with the remaining anti-TB drugs. LFTs, including assessments of aspartate transaminase (AST), alanine aminotransferase (ALT), total bilirubin, and alkaline phosphatase (ALP) levels, were performed at each visit during outpatient follow-up.

### DILI classification

DILI cases (Supplementary Table 1) were determined based on the following criteria established by DILI Expert Working Group^18^: (1) alanine aminotransferase (ALT) ≥ fivefold the upper limit of normal (ULN), (2) alkaline phosphatase (ALP) ≥ twofold the ULN, and (3) alanine transaminase (ALT) ≥ threefold the ULN and total bilirubin ≥ twofold the ULN. Cases were further assessed using Roussel Uclaf Causality Assessment Method (RUCAM) by two or more independent experts (JGY, YKG, and SHK) to confirm AT-DILI, and only patients with an RUCAM scale of probable or highly probable (score ≥ 6) were included. Treatment-tolerant controls were selected based on liver enzyme levels within reference ranges. To reduce the potential for selection bias for treatment-tolerant controls, we also included a population control group (Korea1K; http://1000genomes.kr/)^19^ in case-control analyses.

### Targeted pharmacogenomic (PGx) sequencing

Genomic DNA samples obtained from 112 participants in the discovery cohort were subjected to sequencing for 380 pharmacogenes (Supplementary Table 2). Genomic DNA was extracted from peripheral blood samples using QIAamp DNA Mini Kit (Qiagen, Hilden, Germany) according to the manufacturer’s protocol. Target panels were produced to cover all the transcript isoforms (Ensemble transcript) and ± 50 bp fragments of nearby junctions using an in-solution hybrid capture method, as described previously (Celemics, Inc., Seoul, Korea)^20^. Total read counts of 10.7 million per sample with a length of 150 bp paired-end reads were generated in 0.87 Mb target regions using the HiSeq 2500 system (Illumina, Inc., San Diego, USA) and mapped to the GrCh37/hg19 reference genome using Burrows–Wheeler Aligner (BWA v0.7.17). The average depth of coverage for target regions was 317 ×, and the fraction of targets above 100 × was 93.6%. The bioinformatics pipeline was constructed following Genome Analysis Tool Kit (GATK) best practices for germline small-nucleotide variant (SNV) discovery using GATK v4.1.4, as described previously^21,22^. Quality control for genotype calls was performed following the GATK genotype refinement workflow. Variants with a genotype quality <20, depth of coverage <20, missing genotypes >10%, or violating the Hardy‒Weinberg equilibrium (*P* < 10^−6^) were filtered out. Functional annotation of the detected variants (Supplementary Table 3) was performed using ANNOVAR with the RefGene, EnsGene, Cytoband, avSNP150, gnomAD v2.1.1, dbNSFP v4.1a, and PharmGKB databases^23,24^.

### Assessment of known candidates: *NAT2*, *CYP2E1*, and *GSTM1*

Candidate markers previously known to be associated with AT-DILI were evaluated for the *NAT2*, *CYP2E1*, and *GSTM1* genes (Supplementary Table 4)^25^. NAT2 acetylator status was determined as previously described: (1) rapid acetylator (RA; *4/*4), (2) intermediate acetylator (IA; *4/*5, *4/*6, *4/*7, *4/*12, and *4/*19), (3) slow acetylator (SA; *5/*6 and *5/*7), and (4) UA (*6/*6, *6/*7, and *7/*7)^11^. The *CYP2E1* genotype was previously classified as c1/c1 (wild-type; WT), c1/c2, or c2/c2; these genotypes indicate *1A/*1A, *1A/*5 and *5/*5, respectively, according to the recently recommended nomenclature, and the *1A/*1A genotype is regarded as the risk genotype based on the marker rs3813867^25^. Given that the *GSTM1* null variant is a copy-number variant (CNV), a read-depth-based CNV detection method was used to distinguish the null genotype, as previously described^20^.

### Pharmacogenome-wide association study (PGxWAS)

Case‒control association studies were conducted on 1760 common PGx variants (minor allele frequency (MAF) ≥ 5%) detected in the discovery cohort (*n* = 112). Sex chromosomes were excluded from the analysis owing to the complexity of the statistical analysis. An ancestry-matched Korean population control (*n* = 1048) was used to increase the statistical power. Whole-genome sequencing data originating from Korean Genome Project (Korea1K) were obtained from the Korean Genomics Center at the Ulsan National Institute of Science and Technology (UNIST)^19^. Variants located in the target regions were extracted for 1048 individuals who participated in the Korea1K dataset after liftover (hg38 to hg19) and quality control processes. Consequently, association analyses were conducted using two different controls (Supplementary Table 5): a treatment-tolerant control (*n* = 77) and a population control (*n* = 1048) using PLINK software v1.9 (https://www.cog-genomics.org/plink/)^26^. Fisher’s exact test was used to calculate *P* values under the Cochran-Armitage trend model. Manhattan plots and QQ plots (Supplementary Fig. 1) were generated using the R package ‘qqman’^27^.

### Rare variant association analysis

Gene-based association analysis was carried out using less frequent functional variants (MAF < 5%, obtained from the gnomAD exome v2.1.1 EAS population frequency). The functional variants included missense, nonsense, frameshift, and splicing variants (within 2 bp at exon junctions), as defined by Ensemble annotation. Gene-based collapsing was used to construct gene sets from these variants. The sequence kernel association test (SKAT), optimized SKAT (SKAT-O), and burden test were applied to the DILI patients and treatment controls using the R package ‘SKAT’^28,29^. Genes with more than two functional variants and *P* < 0.05 are listed in Supplementary Table 6.

### *NAT2* and *ATP7B* genotyping

Sequencing of the *NAT2* gene in the discovery cohort indicated that the following five *NAT2* haplotypes could be used to distinguish among Koreans: rs1041983 (c.282C>T, p.Y94Y; C___8684085_20), rs1801280 (c.341T>C, p.I114T; C___1204093_20), rs1799930 (c.590G>A, p.R197Q; C___1204091_10), rs1208 (c.803A>G, p.K268R; C____572769_20), and rs1799931 (c.857G>A, p.G286E; C____572770_20). One patient harbored the rare variant rs1805158 (c.190C>T, p.R64W), which did not affect the acetylator phenotype. Therefore, *NAT2* and *ATP7B* genotyping was performed for the above five single-nucleotide polymorphisms (SNPs) and the *ATP7B* lead SNP rs1061472 (c.2495A>C, p.K832R) using genomic DNA samples acquired from 165 participants in the replication cohort. A TaqMan assay (Thermo Fisher Scientific, Massachusetts, USA) was used for genotyping using the ABI PRISM 7900HT real-time PCR system (Applied Biosystems, Foster City, CA, USA).

### Tissue-specific gene expression data

Tissue-specific gene expression data were analyzed via the Genotype-Tissue Expression (GTEx) portal (https://www.gtexportal.org/). The 19 variants in the four candidate genes (*ATP7B*, *NAT1*, *PROM2*, and *SLCO2B1*; Supplementary Table 5) were investigated in GTEx to assess whether there is a variant associated with gene expression. Tissue and isoform expression data for the *NAT1* gene and the variant rs7845127 were obtained. The sQTL violin plot for the variant (rs7845127) was generated from the 208 genotyped liver tissue samples using GTEX Analysis Release V8 data (dbGaP Accession phs000424.v8.p2; Supplementary Fig. 6).

### Cell lines

Details on the cell and molecular experimental procedures used are described in the Supplementary Materials and Methods. Human hepatocellular carcinoma (HepG2) cells (HB-8065) and human embryonic kidney (HEK) 293 T cells (CRL-3216) were obtained from the American Type Culture Collection (ATCC, USA). SNU387 cells (CRL-2237) were obtained from the Korean Cell Line Bank (KCLB, Republic of Korea).

### Generation of gene knockout and knockdown cells

The CRISPR‒Cas9 system was used to generate *ATP7B* knockout (KO) HepG2 cells. The lentiCRISPRv2 vector (Addgene #52961) was digested with BsmBI and ligated with gRNAs targeting exons 9 and 17 of the *ATP7B* gene. To generate a stable *NAT2* knockdown construct in SNU387 cells using lentiviral vectors, HEK293T cells were plated at a density of approximately 7.0 × 10^5^ cells in 5 mL of medium in a 6 cm tissue culture plate one day before transfection. NAT2 short hairpin RNA (shRNA) (TRCN0000034910) and the empty pLKO.1 vector were obtained from the human RNAi Consortium (TRC) library. The sequence information of the gRNA, shRNA, and primers used in this study is listed in Supplementary Table 10.

### NAT2 and ATP7B overexpression

We examined the effects of *NAT2* and *ATP7B* gene supplementation by introducing pCMV3 vectors containing cDNA purchased from Sino Biological (#HG11247-CF and #HG17426-UT, respectively). Mutant plasmids encoding ATP7B-R832 and NAT2*7 (G286E) were generated by PCR-based site-directed mutagenesis. Flag-tagged NAT2 plasmids were transfected into HepG2 cells, and the effects of NAT2 overexpression were compared with those of empty vector transfection. We also exogenously expressed the ATP7B WT (K832), ATP7B mutant (R832), NAT2 WT (*4) and NAT2 mutant (*7) alleles in *ATP7B* KO HepG2 cells to compare their activities.

### Fluorescence-activated cell sorting (FACS)

FACS analyses were used to study the effects of various concentrations of INH (Sigma‒Aldrich I3377, St. Louis, USA), copper (II) chloride (CuCl_2_, Sigma‒Aldrich 222011, St. Louis, USA), and carbonyl cyanide 4-(trifluoromethoxy)-phenylhydrazone (FCCP, Sigma‒Aldrich C2920, St. Louis, USA) on WT or *ATP7B* KO HepG2 cells.

### Western blotting

HepG2 cells, carrying WT or mutant plasmids, were cultured and treated with INH and CuCl_2_ before lysis and protein extraction. The proteins were then separated, transferred to membranes, and subjected to western blotting using primary and HRP-conjugated secondary antibodies, after which the bands were visualized through an enhanced chemiluminescence (ECL) detection reagent. For oligomerization studies, the HMGB1 protein was treated with CuCl_2_ and H_2_O_2_ and analyzed under nonreducing conditions; the details of the antibodies used are provided in Supplementary Table 11.

### DNA fragmentation assay

DNA fragmentation was detected using an Apoptotic DNA Ladder Kit (Roche, 11835246001, Germany) according to the manufacturer’s instructions.

### Viability measurements

Cell viability (%) was calculated using absorbance data following the equation (A_Treated_ - A_Blank_)/(A_Control_ - A_Blank_) × 100. The lethal dose (LD50) was calculated from the best-fit dose-response curve using the least-squares method via the following equation: Y = Bottom + (Top-Bottom)/(1 + 10^((LogLD50 - X)*Hillslope).

### Statistical analysis

Comparisons of two groups were carried out using the Mann‒Whitney U test. Odds ratios (ORs) and 95% confidence intervals (CIs) were calculated from contingency tables based on Fisher’s exact test. The Benjamini–Hochberg method was used to determine the false discovery rate (FDR) for multiple corrections. All statistical analyses of genetic data were conducted using R v3.6 software (*R* Foundation for Statistical Computing, Vienna, Austria). Cell and molecular data were analyzed via Student’s *t*-test, multiple *t*-tests with FDR correction, or analysis of variance followed by Tukey’s multiple comparison test, as appropriate, using the GraphPad Prism software package (v8.4.2). A significance level of *P* < 0.05 was used throughout the analyses.

Please see the Supplementary Information for detailed information on the materials and processes used in this study.

## Results

### Pharmacogenes and patient characteristics

**Fig. 1:**
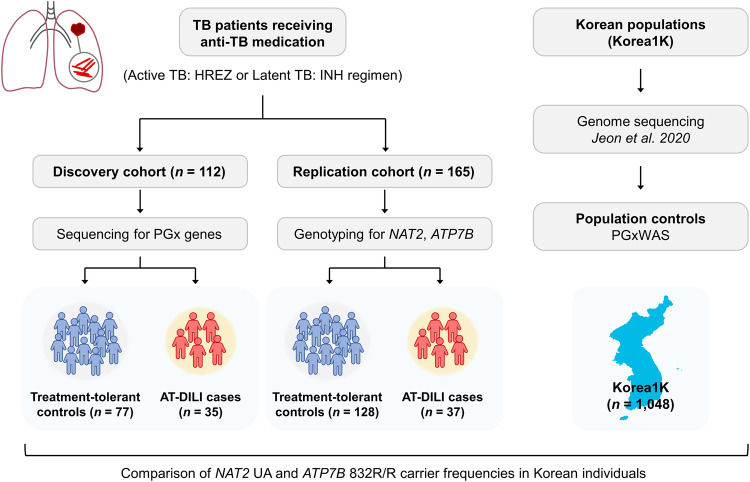
Study cohorts and workflow. A schematic depiction illustrating the study cohorts and workflow. The study comprised two primary cohorts, discovery and replication cohorts, both of which consisted of patients who received anti-TB medications. In the discovery cohort, sequencing was conducted for a comprehensive set of 380 pharmacogenes. Subsequently, the replication cohort was genotyped specifically for the *NAT2* and *ATP7B* genes. Furthermore, we included a Korean population control group, which was sourced from the Korean Genome Project dataset, encompassing genetic data for 1048 healthy individuals who had undergone genome sequencing.

In the present study, we aimed to investigate the relationship between pharmacogenetic variants and the development of DILI (Fig. 1). For this purpose, a sequencing study was conducted on 380 pharmacogenes (PGxs) associated with drug absorption, distribution, metabolism, and excretion (ADME), as well as pharmacodynamics, in 112 participants enrolled in the discovery cohort. PGx genes were selected based on comprehensive literature reviews and information from the PharmGKB database (https://www.pharmgkb.org/) (Supplementary Table 2). The discovery cohort consisted of 35 cases of DILI, including 33 hepatocellular and 2 mixed-type DILI. Additionally, 77 treatment-tolerant controls diagnosed with active TB and treated with the standard regimen were included. To validate our findings, we established a replication cohort comprising patients with both active TB (*n* = 101) and latent TB (*n* = 64) who were treated with the standard TB regimen and INH monotherapy, respectively, and conducted *NAT2* and *ATP7B* genotyping. Within the replication cohort, we identified 37 patients with DILI, as categorized into 29 hepatocellular, 5 mixed-type, and 3 cholestatic-type cases. In total, the combined cohort consisted of 72 DILI patients of 277 patients, with hepatocellular types comprising the majority (86.1%) (Supplementary Table 1). In both the discovery and replication cohorts (Table 1), no significant differences were observed between the case and control groups in terms of sex or age. However, it was notable that the frequency of DILI was greater in patients with active TB (28.7%) than in those with latent TB (12.5%) in the replication cohort (*P* = 0.021). This difference can be partially attributed to variations in drug regimens between the active TB group (HREZ combination therapy) and the latent TB group (INH monotherapy).

### Pharmacogenomic variants associated with AT-DILI

**Fig. 2:**
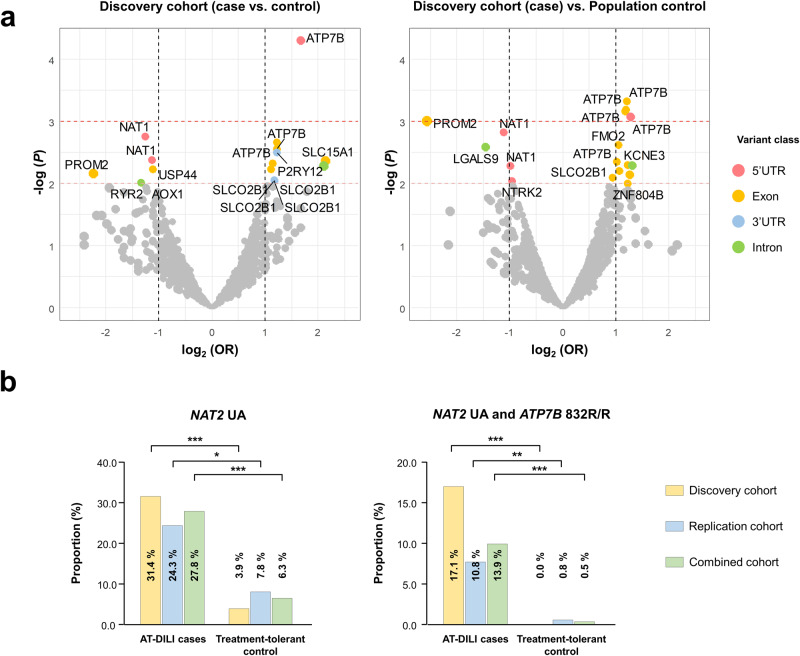
Discovery of pharmacogenetic variants associated with AT-DILI. **a** Volcano plots showing the log2-transformed odds ratio (OR) on the x-axis and -log *P* value on the y-axis of the pharmacogenome-wide association study (PGxWAS) conducted in two different controls. The left panel shows the results from the comparison of 35 AT-DILI patients vs. 77 treatment-tolerant controls, and the right panel shows the results from the comparison of 35 AT-DILI patients vs. 1048 population controls. The *ATP7B* gene consistently showed the strongest signal in these comparisons. The orange and red dashed lines represent significance thresholds of *P* < 0.01 and *P* < 0.001, respectively. **b** Higher frequencies of *NAT2* UAs were observed in AT-DILI patients than in treatment-tolerant controls in both the discovery (*n* = 112) and replication (*n* = 165) cohorts (OR 5.6 [2.5–13.2], *P* = 7.2 × 10^−6^). Furthermore, the combination of *NAT2* UAs with *ATP7B* 832R/R occurred more frequently in AT-DILI patients (OR 32.5 [4.5–1423], *P* = 7.5 × 10^−6^; *P < 0.05; **P < 0.01; ***P < 0.001).

In the discovery cohort, we conducted sequencing analysis and identified a total of 4127 variants in the target regions of 380 pharmacogenes. Notably, 373 of these variants are novel and not reported in the dbSNP 150 database. These novel variants hold potential significance in influencing individual drug responses, as indicated in Supplementary Table 3. For our pharmacogenome-wide association study (PGxWAS), we analyzed 35 patients with DILI and two sets of controls: 77 treatment-tolerant individuals and 1048 population controls (Fig. 2). This analysis revealed several candidate genes, namely, *ATP7B*, *NAT1*, *PROM2*, and *SLCO2B1*, with the strongest association signal observed for the *ATP7B* gene (Fig. 2a, Supplementary Table 5). Our findings were supported by the quantile-quantile (QQ) plot, which demonstrated significant inflation signals compared to the population control (Supplementary Fig. 1). Specifically, we observed a potential association between the *ATP7B* K832R (rs1061472) variant, which is known to alter ATP7B properties according to previous studies^30,31^. Additionally, we assessed genes with known associations^12,13^ and found a significant association between NAT2 UA status and AT-DILI (OR 11.0 [2.6–66.7], *P* = 1.4 × 10^−4^). However, no associations were found with nonfunctional alleles of the *CYP2E1* or *GSTM1* gene (Supplementary Table 4). Moreover, we performed rare variant analyses using SKAT, SKAT-O, and Burden tests, but no significant associations with AT-DILI in other pharmacogenes were observed after correcting for multiple comparisons (Supplementary Table 6).

Based on these results, we identified *NAT2* and *ATP7B* genotypes as potential contributors to the risk of AT-DILI. To further investigate these genotypes, we conducted comprehensive analysis of both the discovery cohort (*n* = 112) and the replication cohort (*n* = 165). The frequency of NAT2 genotypes in our cohort aligned with previous reports on 1,000 Koreans and the Korea1K group (Supplementary Table 7)^19,32^. Consistent with prior findings, NAT2 UAs were associated with an increased risk of AT-DILI in the combined cohort (OR 5.6 [2.5–13.2], *P* = 7.2 × 10^−6^; Table 2)^9–11^. Interestingly, we observed a far greater incidence of co-occurring NAT2 UAs and ATP7B 832 R/R homozygosity in AT-DILI patients (13.9%, 10/72) than in treatment-tolerant controls (0.5%, 1/205; *P* = 0.017; Fig. 2b). While *ATP7B* 832R/R homozygosity alone exhibited a modest effect, combination with *NAT2* UAs (resulting in co-occurrence of NAT2-ATP7B risk genotypes) significantly increased risk (OR 32.5 [4.5–1,423], *P* = 7.5 × 10^−6^). In the discovery cohort, six of 35 AT-DILI patients (17.1%) had these genotypes, but none of the 77 treatment-tolerant controls (0.0%) had these genotypes (OR > 66.4, *P* = 2.2 × 10^−16^). Similarly, in the replication cohort, four of 37 AT-DILI patients (10.8%) and one of the 128 treatment-tolerant controls (0.8%) carried these genotypes (OR 15.1 [1.43–760], *P* = 0.009). Overall, *NAT2*-*ATP7B* risk genotypes were present in 13.9% of all AT-DILI patients (*n* = 72), which was significantly greater than the corresponding proportions in the treatment-tolerant controls (*n* = 205; 0.5%) and population controls (*n* = 1048; 1.8%, Supplementary Table 8).

**Table 2:** High frequencies of co-occurring *NAT2* and *ATP7B* risk genotypes in patients with anti-tuberculosis drug-induced liver injury.

Risk Genotypes		*NAT2* UA		*ATP7B* 832 R/R		*NAT2* UA + *ATP7B* 832 R/R	
		*n*	%	*n*	%	*n*	%
**Discovery cohort (*n*** = **112)**	Case	11	31.4	11	31.4	6	17.1
	Control	3	3.9	10	13	0	0
	OR [95% CI]	11.02 [2.62–66.67]		3.04 [1.03–9.13]		>66.4	
	*P* value	1.4 × 10^−4^		0.035		6.8 × 10^−4^	
**Replication cohort (*n*** = **165)**	Case	9	24.3	9	24.3	4	10.8
	Control	10	7.8	22	17.2	1	0.78
	OR [95% CI]	3.75 [1.23–11.41]		1.54 [0.56–3.98]		15.06 [1.43–760]	
	*P* value	0.015		0.34		9.3 × 10^−3^	
**Combined cohort (*n*** = **277)**	Case	20	27.8	20	27.8	10	13.9
	Control	13	6.3	32	15.6	1	0.48
	OR [95% CI]	5.63 [2.48–13.22]		2.07 [1.03–4.10]		32.45 [4.46–1424]	
	*P* value	7.2 × 10^−6^		0.034		7.5 × 10^−6^	
**Population control (*n*** = **1,048)**		129	12.3	147	14	19	1.8
	OR [95% CI]^a^	2.73 [1.50–4.84]		2.35 [1.29–4.15]		8.70 [3.46–20.67]	
	*P* value^a^	8.8 × 10^−4^		0.003		4.8 × 10^−6^	

^a^Comparison with the combined cohort.

### Synergistic toxicity caused by INH and Cu

**Fig. 3:**
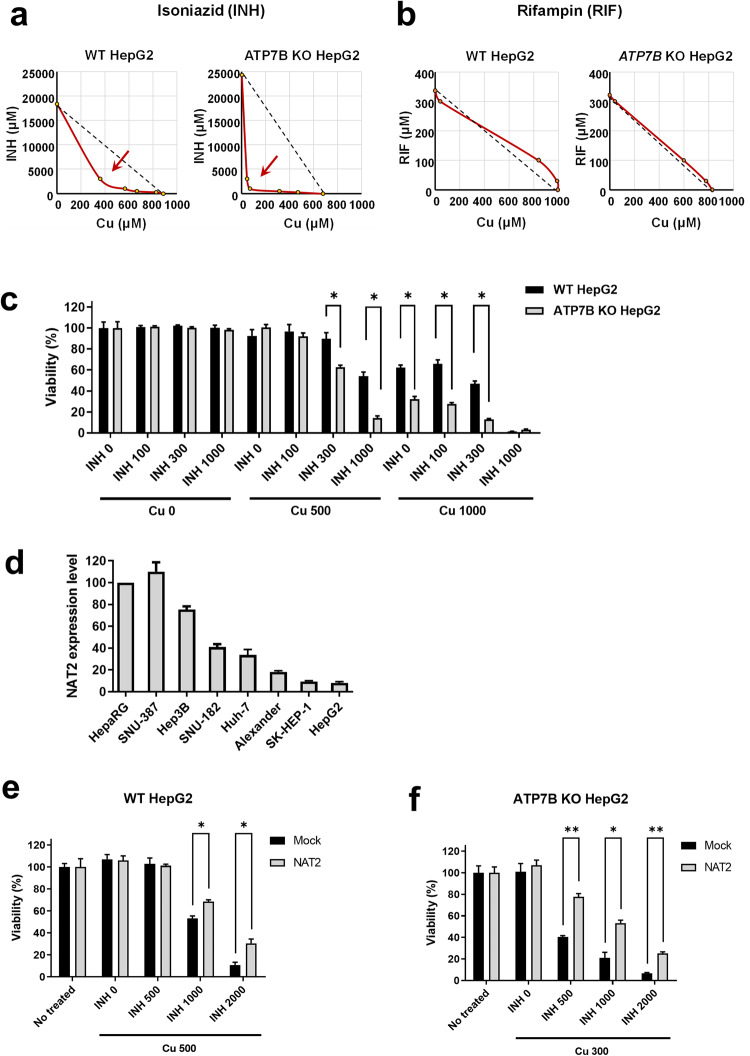
Synergistic toxicity between isoniazid (INH) and copper (Cu) is regulated by ATP7B and NAT2 activities. Assessment of drug synergism between copper (Cu) and antituberculosis drugs. In HepG2 cells, CC_50_ (the 50% cytotoxic concentration) values were measured for various combinations of **a** isoniazid (INH) or **b** rifampin (RIF) with Cu for 48 h. Isobolograms of CC_50_ values for INH-Cu and RIF-Cu cotreatments are shown. The dashed lines indicate the additive isobole (no interaction). A toxic synergistic effect between INH and Cu was observed but not between RIF and Cu. The toxic synergistic effect was more pronounced in *ATP7B* knockout (KO) cells. **c** Cell viability assay. The INH-induced cell death rate in the presence of Cu (for 24 h) was significantly greater in *ATP7B* KO cells than in wild-type (WT) cells. **d** Expression of NAT2 mRNA in eight human liver-derived cell lines was examined via quantitative reverse transcription PCR (*n* = 3). Among the cell lines tested, SNU387 cells exhibited the highest expression of NAT2, whereas HepG2 cells exhibited the lowest. **e**, **f** Exogenous expression of NAT2 attenuates the toxicity caused by treatment with INH and Cu (for 24 h) in HepG2 cells. The effect of exogenous NAT2 expression on cell survival was more pronounced in *ATP7B* KO cells (**c**, *n* = 5) than in WT cells (**b**, *n* = 5). Bar graph data are shown as the mean ± SEM. *P < 0.05 according to multiple t tests with FDR correction.

To identify the underlying molecular mechanisms of *NAT2* and *ATP7B-*associated AT-DILI, we first evaluated the synergistic cytotoxicity between AT medications (INH and RIF) and Cu by measuring the viability of hepatocyte-derived HepG2 cells. Notably, isobolographic analyses of CC_50_ (cytotoxic concentration 50) revealed that Cu has a toxic synergism with INH but not with RIF (Fig. 3a, b).

Next, the effects of NAT2 and ATP7B on INH-Cu cytotoxicity were evaluated via gene deletion and supplementation experiments. We generated *ATP7B* KO HepG2 cells using the CRISPR‒Cas9 system and then exogenously expressed WT (K832) and mutant (R832) ATP7B in the KO cells using plasmids containing the codon-optimized ATP7B sequence that prevents guide RNA-mediated degradation (Supplementary Fig. 2a, b). Importantly, *ATP7B* KO strongly augmented the synergistic effect of INH-Cu on toxicity according to isobolographic analysis (Fig. 3a); thus, INH-induced cell death in the presence of Cu was significantly greater in *ATP7B* KO cells than in WT cells (Fig. 3c).

Since HepG2 cells barely express detectable levels of NAT2 (Fig. 3d), we analyzed the effects of NAT2 on INH-Cu toxicity via gene supplementation. Exogenous NAT2 expression partially mitigated INH-Cu-induced cell death in WT HepG2 cells (Fig. 3e). Notably, the effect of exogenous NAT2 expression was more prominent in *ATP7B* KO HepG2 cells (Fig. 3f), indicating that simultaneous reductions in NAT2 and ATP7B activities facilitate INH-Cu toxicity.

**Fig. 4:**
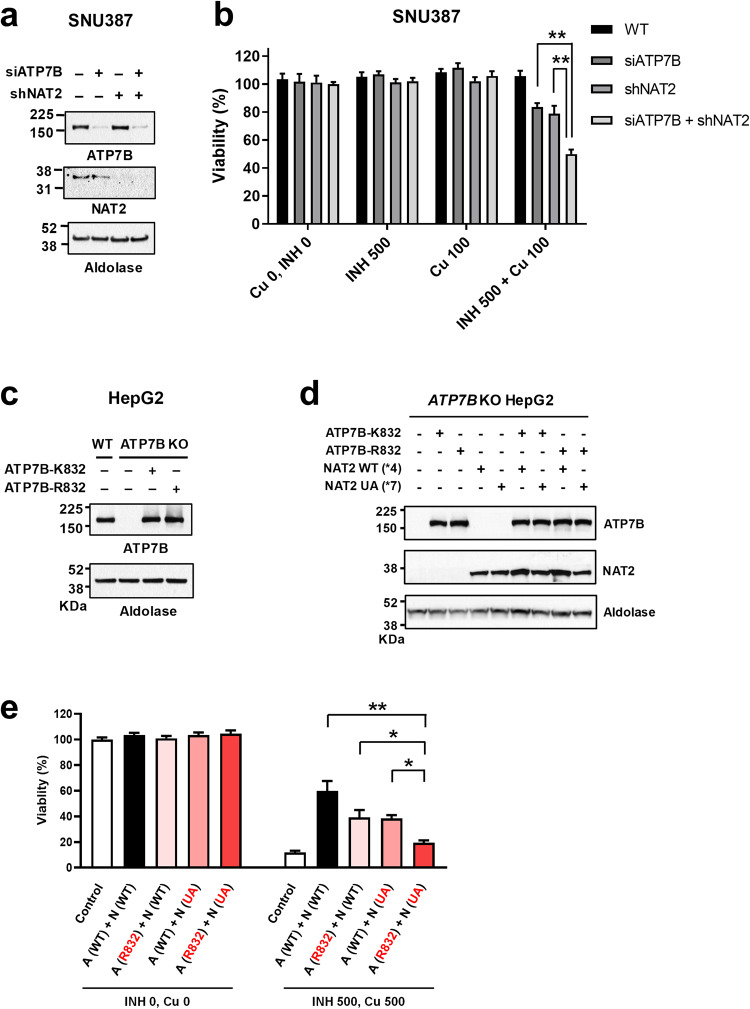
Reduced activity of NAT2 and ATP7B augments isoniazid (INH)-copper (Cu) toxicity in SNU387 and HepG2 liver-derived cells. **a**, **b** Toxicity of INH-Cu (for 48 h) was assayed in SNU387 cells with knockdown of *NAT2* and *ATP7B*. Gene knockdown of *NAT2* was achieved via stable expression of shRNAs against *NAT2*, and that of *ATP7B* was achieved through transient transfection with siRNAs against *ATP7B*. Western blotting images verifying the effects of *NAT2* and *ATP7B* knockdown (**a**). Single knockdown of either *NAT2* or *ATP7B* partially reduced cell survival in the presence of INH and Cu (for 48 h), and double knockdown of *NAT2* and *ATP7B* further augmented INH-Cu toxicity (**b**, *n* = 4). Bar graph data are shown as the mean ± SEM. *P < 0.05, **P < 0.01 by multiple t tests with FDR correction. Effects of exogenously supplementing ATP7B and NAT2 with the wild-type (WT) or mutant protein in *ATP7B* KO cells were examined. An example of immunoblotting for native and exogenous ATP7B expression is shown in **c**, and a summary of the densitometric analysis is shown in Supplementary Fig. 2b. Coexpression of WT and mutant ATP7B and NAT2 proteins in *ATP7B* KO cells via plasmid transfection was confirmed through immunoblotting (**d**). A cell viability assay was performed on cells treated with INH and Cu for 48 h **e**. Exogenous expression of WT ATP7B (K832) or NAT2 (*4) led to alleviation of INH-Cu toxicity. Cell survival effects were greatly diminished in cells expressing the mutant ATP7B-R832 and NAT2-UA (*7B) proteins. A (WT), ATP7B wild-type; A (R832), ATP7B-R832; N (WT), NAT2 wild-type; N (UA), NAT2*7B. Bar graph data are shown as the mean ± SEM (*n* = 5–8). *P < 0.05 by ANOVA followed by Tukey’s multiple comparison test. Concentrations of INH and Cu are in μM.

Because SNU-387 cells endogenously express ATP7B and exhibited the highest expression levels of NAT2 among the examined hepatocyte-derived cells (Figs. 3d and 4a), we evaluated the effects of ATP7B and NAT2 silencing in these cells. NAT2-downregulated cells were generated by stably expressing shRNAs targeting NAT2. Gene knockdown of *ATP7B* was achieved in these cells by transient treatment with siRNAs against *ATP7B* (Fig. 4a and Supplementary Fig. 2c, d). Single knockdown of either *NAT2* or *ATP7B* partially reduced cell survival in the presence of INH and Cu (Fig. 4b). Notably, double knockdown of *NAT2* and *ATP7B* further increased susceptibility to INH-Cu toxicity (Fig. 4b), which provides a basis for the increased risk of AT-DILI in individuals with high-risk *NAT2* and *ATP7B* genotypes.

Then, we examined the effects of exogenously supplementing ATP7B and NAT2 with the WT or mutant protein in *ATP7B* KO HepG2 cells (Fig. 4c–e). The toxicity of INH-Cu (INH 500 μM, Cu 500 μM) was substantially rescued by exogenous expression of the WT-ATP7B (K832) and WT-NAT2 (*4) proteins (Fig. 4e, black bar). This cell survival effect of ATP7B was partially reduced in cells expressing mutant ATP7B-R832 (Fig. 4e), which is consistent with previous reports that ATP7B-R832 is a hypomorphic mutant^30,31^. Notably, cell survival was greatly diminished in cells expressing the mutant ATP7B-R832 and NAT2-UA (*7) proteins, indicating that hepatocytes with mutant *ATP7B* and *NAT2* alleles are highly vulnerable to INH-Cu toxicity.

### INH and Cu evoke mitochondrial injury

**Fig. 5:**
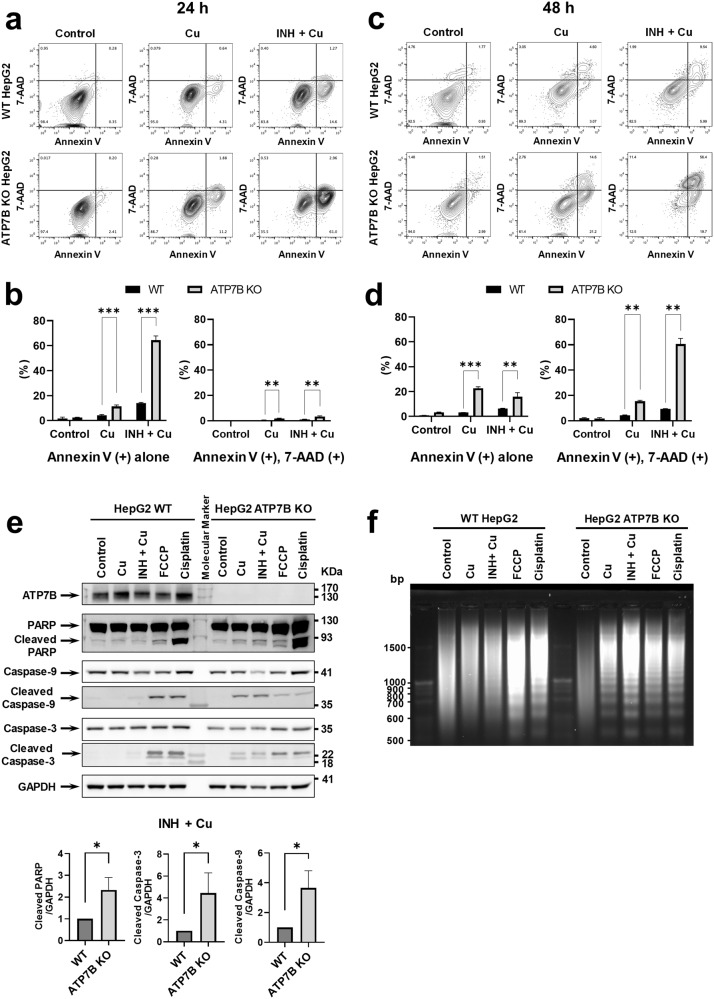
Isoniazid (INH) and copper (Cu) induce apoptosis in HepG2 cells. **a-d** Examples of FACS dot plots constructed for HepG2 cells treated with INH (1000 µM) or Cu (500 µM) for 24 h or 48 h are shown in **a** and **c**, respectively. Summarized results of multiple experiments are presented in **b** and **d** (*n* = 3). Treatment with INH-Cu for 24 h strongly increased the percentage of annexin V-positive *ATP7B* KO cells, and a longer period of INH and Cu incubation (48 h) produced annexin V and 7-AAD double-positive cells. **e** Western blot analysis of apoptosis markers in HepG2 cells treated with INH or Cu for 24 h. Summarized results of multiple experiments are presented in the lower panels (*n* = 3). **f** Results of an apoptotic DNA fragmentation assay. Increased DNA fragmentation was noted in *ATP7B* KO cells treated with Cu alone or INH-Cu. Identical results were obtained in four independent experiments. Bar graph data are shown as the mean ± SEM. *P < 0.05, **P < 0.01, ***P < 0.001 by multiple t tests with FDR correction. FCCP (25 µM) and cisplatin (40 µM) were used as positive controls.

We next examined the cellular mechanisms responsible for INH-Cu toxicity. Initially, cell death patterns were analyzed via FACS analyses in HepG2 cells stained with annexin V and 7-AAD. Control experiments in HepG2 cells treated with cell injury-inducing agents, such as FCCP and H_2_O_2_, showed that *ATP7B* KO did not induce differences in intrinsic cell survival (Supplementary Fig. 3). In addition, treatment with INH alone at concentrations up to 1000 µM did not significantly induce cell death or mitochondrial injury (Supplementary Fig. 4). Subsequently, we investigated the toxicity mechanisms associated with Cu alone or INH-Cu treatment (Fig. 5). Treatments with Cu alone up to 500 µM elicited no or mild cell toxicity. Notably, treatment with INH and Cu for 24 h strongly increased the number of annexin V-positive *ATP7B* KO cells (Fig. 5a, b), and a longer period of INH and Cu incubation (48 h) eventually produced annexin V and 7-AAD double-positive cells (Fig. 5c, d). Taken together, these findings suggest that apoptotic mechanisms are primarily involved in INH-Cu cytotoxicity. Consequently, western blot analyses of apoptosis markers showed that cotreatment with INH and Cu for 24 h triggered a series of apoptotic signals, such as increases in levels of the cleaved forms of PARP, caspase-3, and caspase-9, in *ATP7B* KO cells (Fig. 5e). A DNA fragmentation assay also indicated that apoptosis-induced DNA fragmentation was increased by INH and Cu treatment, particularly in *ATP7B* KO cells (Fig. 5f).

**Fig. 6:**
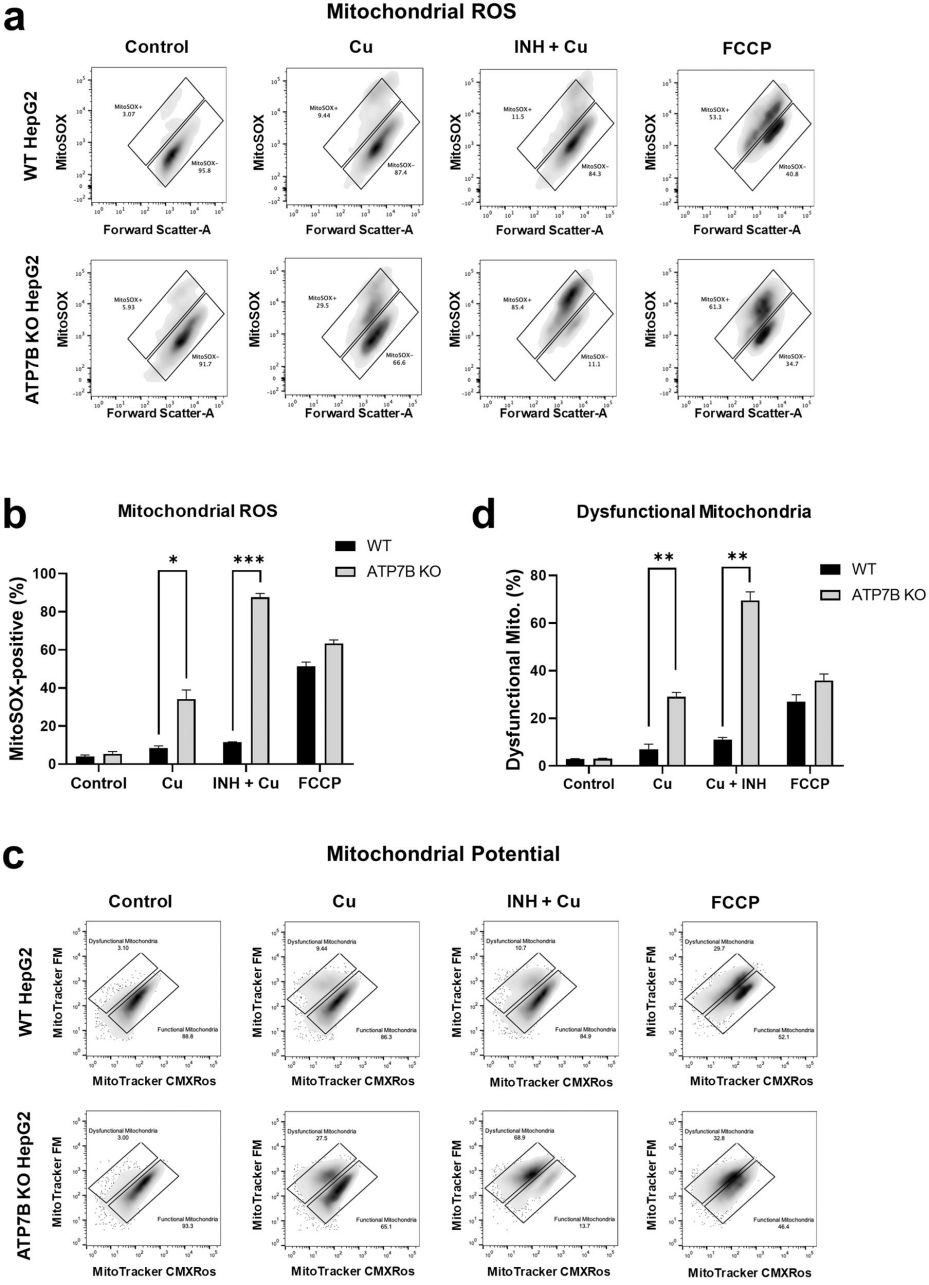
Isoniazid (INH) and copper (Cu) induce mitochondrial dysfunction in HepG2 cells. Measurements of mitochondrial ROS generation using FACS analyses with MitoSOX. Examples of FACS dot plots are shown (**a**), and results of multiple experiments are presented in (**b**, *n* = 3). The percentage of MitoSOX-positive cells was greatly increased in *ATP7B* KO cells treated with INH (1000 µM) or Cu (500 µM) for 24 h. Measurement of functional mitochondria (%) using the MitoTracker system. MitoTracker CMXRos stains mitochondria in live cells, and its relative accumulation compared to MitoTracker FM depends on membrane potential. Examples of FACS dot plots are shown (**c**), and results of multiple experiments are presented in (**d**, *n* = 3). The percentage of dysfunctional mitochondria was greatly increased in *ATP7B* KO cells treated with INH or Cu for 24 h. Bar graph data are shown as the mean ± SEM. *P < 0.05, **P < 0.01, ***P < 0.001 by multiple t tests with FDR correction. FCCP (25 μM) was used as a positive control.

Mitochondria play a central role in apoptotic cell death^33^, and dysfunction of these organelles is known to be associated with diverse types of liver injury, including INH-induced DILI^34^. While healthy mitochondria spontaneously produce reactive oxygen species (ROS) as a byproduct via the electron transport chain reaction, damage to mitochondria might cause overproduction of ROS, which, in turn, may trigger cellular signaling that can induce cell death in various ways. Notably, FACS analyses using probes responsive to mitochondrial ROS (MitoSOX) and mitochondrial membrane potentials (MitoTracker CMXRos) revealed that INH and Cu treatment strongly increased both mitochondrial ROS generation (Fig. 6a, b) and the number of dysfunctional mitochondria (Fig. 6c, d) in *ATP7B* KO cells. Taken together, these results imply that accumulation of Cu in *ATP7B* KO HepG2 cells evokes synergistic toxicity with INH via mitochondrial injury, which leads to apoptotic cell death.

Nevertheless, other types of cell death pathways, such as necrosis and pyroptosis, did not appear to play major roles in INH-Cu toxicity. For example, treatment of ATP7B KO cells with INH and Cu resulted in a slight increase in the level of cellular lipid peroxidation, which seems to be linked to excessive production of mitochondrial ROS. However, the increase in lipid peroxidation induced by INH and Cu was only 13% of that induced by H_2_O_2,_ causing necrotic cell death (Supplementary Fig. 5a, b). In addition, INH-Cu treatment neither evoked activation of caspase-1 or Gasdermin D (Supplementary Fig. 5c) nor oligomerization of HMGB1 (Supplementary Fig. 5d), implying that pyroptotic mechanisms are not directly involved in INH- and Cu-induced cell death in HepG2 cells.

## Discussion

The current understanding of the mechanisms underlying AT-DILI remains incomplete. Previous studies, including meta-analyses and GWASs, have demonstrated an association between *NAT2* UA status and an elevated risk of AT-DILI^9–11^. However, the specific pathways influencing the risk of AT-DILI have yet to be fully elucidated. In this study, we investigated the genetic variants and molecular mechanisms that may predispose individuals to greater AT-DILI susceptibility. We found that individuals with genetic variations in the Cu transporter *ATP7B* gene and in the INH-metabolizing enzyme *NAT2* gene are at a greater risk of developing AT-DILI. Specifically, these two genes exhibited strong gene-gene interactions in AT-DILI. The presence of an *ATP7B* variant significantly amplifies the risk of developing AT-DILI when combined with the *NAT2* UA genotype, as more than 90% of individuals with these combined genotypes are likely to develop AT-DILI. By conducting additional studies, we further investigated the underlying mechanisms involved. Our results suggest that Cu exerts synergistic toxicity with INH, potentially through mitochondrial dysfunction. These findings shed light on the mechanism of INH-induced hepatotoxicity and may help to identify patients at high risk of developing AT-DILI to prevent adverse drug reactions during TB management.

In addition to the *NAT2* and *ATP7B* variants, our study identified several PGx variants that might contribute to variations in individual drug responses. Notably, among these are common variants of the *NAT1*, *PROM2*, and *SLCO2B1* genes (Supplementary Table 5), as well as rare variants of the *GSTM2*, *ADH1B*, *HMOX2*, *SCN1A*, and *TNFRSF4* genes (Supplementary Table 6). Although these differences did not reach statistical significance in our analysis, conducting further investigations with a larger cohort may provide statistical power to validate their involvement in AT-DILI. Of particular interest is the *NAT1* variant (rs7845127), which we observed to be a splicing quantitative trait locus (sQTL) potentially associated with increased expression of a liver-specific isoform (ENST00000517441.5) of *NAT1* (Supplementary Fig. 6). Considering the established role of N-acetyltransferases in INH metabolism, upregulation of NAT1 expression might reduce the risk of AT-DILI, which aligns with the findings observed in our study (odds ratio 0.42–0.51; Supplementary Table 5).

In this study, we employed strict criteria to include only patients with apparent DILI who met the designated threshold of RUCAM score of 6 or higher. Patients with minor elevations in liver function tests were excluded from the discovery cohort. By implementing these stringent criteria, we aimed to ensure the accuracy and reliability of our findings. To minimize potential selection bias, we further conducted case‒control analysis utilizing a population control group (Korea1K, *n* = 1048). Although the number of DILI patients (*n* = 72) and carriers of the NAT2-ATP7B high-risk genotype (*n* = 10, 13.9%) in our study cohort might appear to be relatively small, their significance becomes apparent when considering the low prevalence (1.8%) of high-risk genotype carriers within the Korean population. These results indicate a meaningful association between the high-risk genotype and AT-DILI, and emphasize the need for larger-scale studies to confirm our findings.

We observed a significantly greater risk of AT-DILI in individuals with *NAT2* UAs and *ATP7B* 832R/R homozygotes (OR 32.5 [4.5–1423], *P* = 7.5 × 10^−6^), while *ATP7B* 832R/R homozygotes exhibited a small independent effect size (vs. treatment-tolerant control, OR 2.07 [1.03–4.10], *P* = 0.034; vs. population control, OR 2.35 [1.29–4.15], *P* = 0.003; Table 2). Interestingly, the combination of NAT2 UA genotypes with ATP7B 832R/R homozygosity strongly improved the PPV (60.0% vs. 18.7%, *P* < 0.001) for AT-DILI prediction without significantly compromising the NPV (96.1% vs. 95.6%, *P* > 0.05) compared to NAT2 UA genotypes alone, as shown in Supplementary Table 9. These findings suggest that the inclusion of *ATP7B* genotypes in pharmacogenetic screening for *NAT2* UAs, in combination with use of a genotype-guided regimen^35^, may further improve the prediction of patient susceptibility to AT-DILI. Furthermore, our analysis revealed a noteworthy observation regarding the frequency of high-risk genotype carriers (those with *NAT2* UAs and *ATP7B* 832R/R homozygosity) in South Asian populations (Supplementary Fig. 7). Analysis of the 1000 Genome database indicated a substantially greater prevalence of these genotypes (7.1%) in the South Asian region, which aligns with the reported high incidence of AT-DILI in these populations^3,5–7^.

Many patients in our cohorts had taken AT medications other than INH. One such medication is RIF, which has been reported to cause mitochondrial injury in hepatocytes^36^. Therefore, it is important to consider the possibility of a more complex mechanism underlying the clinical situation. However, our findings revealed that Cu exhibited strong synergistic toxicity with INH (Fig. 3a) but that only additive effects were observed with RIF (Fig. 3b). This observation provides further evidence that INH is primarily responsible for NAT2- and ATP7B-associated AT-DILI. One potential mechanism that may explain this phenomenon is formation of an INH-Cu complex^37,38^. Previous studies have reported that Cu complexes formed with INH and its derivatives possess a strong affinity for DNA binding and display cytotoxic properties, which may contribute to INH-induced hepatotoxicity^39–41^.

In hepatocytes, ATP7B plays a pivotal role in regulating Cu homeostasis^42^. Biallelic mutations in the *ATP7B* gene are known to cause Wilson’s disease, which is characterized by accumulation of intracellular Cu and manifestation of liver symptoms such as acute hepatitis, hepatomegaly, jaundice, and cirrhosis^43^. While the *ATP7B* K832R variant (rs1061472) is generally considered benign in Wilson’s disease, the ATP7B K832R (rs1061472) polymorphism has been shown to alter ATP7B properties in vitro and in vivo, affecting its trafficking and transporting activities as well as its serum Cu levels in humans^30,31,44^. Our study suggests that individuals with Wilson’s disease or ATP7B-defective alleles who also harbor *NAT2* UAs may be at increased risk of INH-induced hepatotoxicity (Fig. 4c). It is possible that the occurrence of INH-induced hepatotoxicity in patients with Wilson’s disease has been underestimated, as diagnosis of DILI often relies on exclusion criteria^45^. In fact, a patient in India diagnosed with Wilson’s disease was reported to develop INH-induced hepatotoxicity^46^. Therefore, caution should be exercised when using INH in patients with Wilson’s disease, particularly individuals with the *NAT2* UA genotype.

In conclusion, our study aimed to identify genetic factors contributing to AT-DILI through targeted PGx sequencing and genotyping. The results revealed that *ATP7B* 832R/R homozygosity increases the risk of AT-DILI in *NAT2* UAs. Experimental analyses demonstrated that ATP7B influences an individual’s susceptibility to INH-induced hepatotoxicity by affecting the synergistic toxicity between Cu and INH, which is mediated through mitochondrial apoptosis. These findings provide novel mechanistic insights into the underlying mechanisms of INH-induced hepatotoxicity and have potential implications for personalized prevention and treatment of AT-DILI, ultimately improving patient care and drug safety in TB management.

### Supplementary information

Supplementary Information

## Data Availability

Targeted sequencing of the PGx panel data can be accessed through the Clinical & Omics Data Archive (CODA; https://coda.nih.go.kr/) under accession number R001218. Any additional information required to reanalyze the data reported in this work is available from the corresponding author upon reasonable request.

## References

[1] Furin J, Cox H, Pai M (2019). Tuberculosis. Lancet.

[2] Andrade RJ (2019). Drug-induced liver injury. Nat Rev Dis Prim.

[3] Tweed CD (2018). Liver toxicity associated with tuberculosis chemotherapy in the REMoxTB study. BMC Med.

[4] Chalasani N (2015). Features and outcomes of 899 patients with drug-induced liver injury: The DILIN Prospective Study. Gastroenterology.

[5] Parthasarathy R (1986). Hepatic toxicity in South Indian patients during treatment of tuberculosis with short-course regimens containing isoniazid, rifampicin and pyrazinamide. Tubercle.

[6] Saha A (2016). Prevalence of hepatotoxicity from antituberculosis therapy: a five-year experience from South India. J Prim Care Community Health.

[7] Devarbhavi H (2021). The Indian network of drug-induced liver injury: etiology, clinical features, outcome and prognostic markers in 1288 patients. J Clin Exp Hepatol.

[8] Huang YS (2002). Polymorphism of the N-acetyltransferase 2 gene as a susceptibility risk factor for antituberculosis drug-induced hepatitis. Hepatology.

[9] Suvichapanich S (2018). NAT2 ultra-slow acetylator and risk of anti-tuberculosis drug-induced liver injury: a genotype-based meta-analysis. Pharmacogenet Genomics.

[10] Suvichapanich S (2019). Genomewide Association Study confirming the association of nat2 with susceptibility to antituberculosis drug-induced liver injury in Thai patients. Antimicrob Agents Chemother.

[11] Nicoletti P (2021). Genetic risk factors in drug-induced liver injury due to isoniazid-containing antituberculosis drug regimens. Clin Pharm Ther.

[12] Kim SH (2009). Genetic polymorphisms of drug-metabolizing enzymes and anti-TB drug-induced hepatitis. Pharmacogenomics.

[13] Chen M, Suzuki A, Borlak J, Andrade RJ, Lucena MI (2015). Drug-induced liver injury: Interactions between drug properties and host factors. J Hepatol.

[14] Chung SJ (2020). Adherence to nine-month isoniazid for latent tuberculosis infection in healthcare workers: a prospective study in a tertiary hospital. Sci Rep.

[15] WHO. Treatment of tuberculosis: guidelines. 4th edn (World Health Organization, 2010). 23741786

[16] Blumberg HM (2003). American Thoracic Society/Centers for Disease Control and Prevention/Infectious Diseases Society of America: treatment of tuberculosis. Am J Respir Crit Care Med.

[17] Saukkonen JJ (2006). An official ATS statement: hepatotoxicity of antituberculosis therapy. Am J Respir Crit Care Med.

[18] Aithal GP (2011). Case definition and phenotype standardization in drug-induced liver injury. Clin Pharm Ther..

[19] Jeon S (2020). Korean Genome Project: 1094 Korean personal genomes with clinical information. Sci Adv.

[20] Han SM (2017). Targeted next-generation sequencing for comprehensive genetic profiling of pharmacogenes. Clin Pharm Ther.

[21] Yoon JG (2021). Unraveling the genomic architecture of the CYP3A Locus and ADME genes for personalized tacrolimus dosing. Transplantation.

[22] Yoon JG (2020). Molecular diagnosis of craniosynostosis using targeted next-generation sequencing. Neurosurgery.

[23] Wang K, Li M, Hakonarson H (2010). ANNOVAR: functional annotation of genetic variants from high-throughput sequencing data. Nucleic Acids Res.

[24] Barbarino JM, Whirl-Carrillo M, Altman RB, Klein TE (2018). PharmGKB: A worldwide resource for pharmacogenomic information. Wiley Interdiscip Rev Syst Biol Med.

[25] Huang YS (2007). Genetic polymorphisms of drug-metabolizing enzymes and the susceptibility to antituberculosis drug-induced liver injury. Expert Opin Drug Metab Toxicol.

[26] Purcell S (2007). PLINK: a tool set for whole-genome association and population-based linkage analyses. Am J Hum Genet.

[27] Turner SD (2018). qqman: an R package for visualizing GWAS results using Q-Q and manhattan plots. J Open Source Softw.

[28] Wu MC (2011). Rare-variant association testing for sequencing data with the sequence kernel association test. Am J Hum Genet.

[29] Lee S (2012). Optimal unified approach for rare-variant association testing with application to small-sample case-control whole-exome sequencing studies. Am J Hum Genet.

[30] McCann CJ (2019). Single nucleotide polymorphisms in the human ATP7B gene modify the properties of the ATP7B protein. Metallomics.

[31] Huster D (2012). Diverse functional properties of Wilson disease ATP7B variants. Gastroenterology.

[32] Lee SY (2002). Complete sequencing of a genetic polymorphism in NAT2 in the Korean population. Clin Chem.

[33] Bock FJ, Tait SWG (2020). Mitochondria as multifaceted regulators of cell death. Nat Rev Mol Cell Biol.

[34] Boelsterli UA, Lee KK (2014). Mechanisms of isoniazid-induced idiosyncratic liver injury: emerging role of mitochondrial stress. J. Gastroenterol Hepatol.

[35] Yoo H (2021). A pilot study to investigate the utility of NAT2 genotype-guided isoniazid monotherapy regimens in NAT2 slow acetylators. Pharmacogenetics Genomics.

[36] Chowdhury A (2006). Mitochondrial oxidative stress and permeability transition in isoniazid and rifampicin induced liver injury in mice. J Hepatol.

[37] Cole A, May PM, Williams DR (1983). Metal binding by pharmaceuticals. Part 3. Copper (II) and zinc (II) interactions with isoniazid. Agents Actions.

[38] Krivis AF, Rabb JM (1969). Cuprous complexes formed with isonicotinic hydrazide. Science.

[39] Divakar S, Vasudevachari MB, Antony A, Easwaran KR (1987). Studies on the interaction of cupric isonicotinohydrazide with DNA. Biochemistry.

[40] Ramadevi P, Singh R, Prajapati A, Gupta S, Chakraborty D (2014). Cu(II) Complexes of Isoniazid Schiff Bases: DNA/BSA Binding and Cytotoxicity Studies on A549 Cell Line. Adv Chem.

[41] Silva PB (2016). In vitro activity of Copper(II) complexes, loaded or unloaded into a nanostructured lipid system, against mycobacterium tuberculosis. Int J Mol Sci.

[42] Polishchuk EV (2014). Wilson disease protein ATP7B utilizes lysosomal exocytosis to maintain copper homeostasis. Dev Cell.

[43] Tao TY, Gitlin JD (2003). Hepatic copper metabolism: insights from genetic disease. Hepatology.

[44] Mercer SW, Wang JB, Burke R (2017). Modeling of the pathogenic effect of copper transporter mutations that cause menkes and wilson diseases, motor neuropathy, and susceptibility to Alzheimer’s disease. J. Biol. Chem..

[45] European Association for the Study of the Liver. (2019). Clinical Practice Guideline Panel, C., Panel, m. & representative, E.G.B. EASL Clinical Practice Guidelines: Drug-induced liver injury. J Hepatol.

[46] Singal P, Punia VP, Lohakare AC, Bansal S (2013). Wilson’s disease unmasked by antitubercular therapy induced liver injury. J Assoc Phys India.

