# Supplementary material for: Synergistic toxicity with copper contributes to NAT2-associated isoniazid toxicity
Source: Exp Mol Med. 2024 Mar 1;56(3):570–82. doi: 10.1038/s12276-024-01172-8 (PMC10984958; doi:10.1038/s12276-024-01172-8)
Supplement: Supplementary file 1 — Supplementary Information [file 12276_2024_1172_MOESM1_ESM.pdf]

Supplementary Information

Synergistic toxicity with copper contributes to NAT2-associated isoniazid toxicity

Jihoon G. Yoon, Dong Geon Jang, Sung-Gyu Cho, Chaeyoung Lee, Shin Hye Noh, Soo Kyung Seo, Jung Woo Yu, Hyeon Woo Chung, KyeoRe Han, Soon Sung Kwon, Dai Hoon Han, Jaeseong Oh, In-Jin Jang, Sang-Hoon Kim, Young-Koo Jee, Hyun Lee, Dong Won Park, Jang Won Sohn, Ho Joo Yoon, Chul Hoon Kim, Jae Myun Lee, Sang-Heon Kim, and Min Goo Lee

< Table of Contents >

Supplementary Materials and Methods..........3

Supplementary References..........9

Supplementary Figures..........10

Supplementary Fig. 1. Manhattan plot and quantile-quantile (QQ) plot of Pharmacogenome (PGx)-wide association study (PGxWAS)..........10

Supplementary Fig. 2. Assessment of gene knockout, knockdown, and supplementation..........11

Supplementary Fig. 3. Knockout of *ATP7B* does not affect the cell injury induced by FCCP and H_2O_2 in HepG2 cells..........12

Supplementary Fig. 4. Isoniazid alone does not induce cell death and mitochondrial injury..........13

Supplementary Fig. 5. Treatment with INH and Cu does not activate necrotic and pyroptotic cell death signals in HepG2 cells..........14

Supplementary Fig. 6. *NAT1* variant (rs7845127) as a splicing quantitative trait locus (sQTL) of liver-specific transcript..15
Supplementary Fig. 7. Global prevalence of *NAT2* and *ATP7B* risk alleles......16

Supplementary Tables……………………………………………………………………17

Supplementary Table 1. Anti-tuberculosis drug-induced liver injury cases included in this study……………………………………………………………..17
Supplementary Table 2. The complete list of 380 target genes…………………..20
Supplementary Table 3. Overview of pharmacogenetic variants detected in the exploratory cohort...22
Supplementary Table 4. Assessment of reported markers associated with anti-tuberculosis drug-induced liver injury 23
Supplementary Table 5. List of variants (MAF > 5%) associated with anti-tuberculosis drug-induced liver injury (AT-DILI) in the discovery cohort (*P* < 0.01)………………………………………………………………………………..24
Supplementary Table 6. Rare variants detected by gene-based association tests 25
Supplementary Table 7. Distribution of *NAT2* acetylator status.......................26
Supplementary Table 8. Co-occurrence of *NAT2* and *ATP7B* risk genotypes in anti-tuberculosis drug-induced liver injury 27
Supplementary Table 9. Predictive values of *NAT2* and *ATP7B* risk alleles in the entire cohort..28
Supplementary Table 10. Sequence information of gRNA, shRNA and primers used in this study..29
Supplementary Table 11. The complete list of antibodies used in this study....30

Supplementary Materials and Methods

Cell lines

Human hepatocellular carcinoma (HepG2) cells (HB-8065) and human embryonic kidney (HEK) 293T cells (CRL-3216) were obtained from the American Type Culture Collection (ATCC, USA). SNU387 cells (CRL-2237) were obtained from the Korean Cell Line Bank (KCLB, Republic of Korea). Cells with a passage of less than 20 were used throughout the experiments. HepG2 and HEK293T cells were maintained in Dulbecco's Modified Eagle's Medium (DMEM; Gibco 11995, CA, USA) supplemented with 10 % (v/v) fetal bovine serum (FBS) and 100 U/ml of penicillin-streptomycin (Gibco 15140163, CA, USA) in a humidified atmosphere of 5 % CO_2 at 37 °C. SNU387 cells were maintained in Roswell Park Memorial Institute medium (RPMI; Capricorn scientific RPMI-A, Ebsdorfergrund, Germany), L-glutamine 300 mg/ml, supplemented with 10 % (v/v) FBS, and 100 U/ml of penicillin-streptomycin in a humidified atmosphere of 5 % CO_2 at 37 °C. THP-1 cells were cultured in RPMI 1640 complete media supplemented with L-Glutamine 2 mM, 10% FBS and 1% penicillin-streptomycin (100U/ml). To differentiate THP-1 monocyte to macrophage, 1.2×10^6 THP-1 cells were initially seeded in RPMI 1640 complete medium including PMA (50 ng/mL) for 24 h. After fully differentiated, THP-1 macrophages were stimulated with LPS (500 ng/ml) for 24 h and ATP (5 mM) for next 2 h to activate inflammation response. The medium was replaced once every 2–3 days, and cells were sub-cultured to maintain the log phase following the recommended methods by the ATCC and KCLB, respectively.

Generation of gene knockout and knockdown cells

The CRISPR-Cas9 system was used to generate *ATP7B* knockout HepG2 cells. The lentiCRISPRv2 vector (Addgene #52961) was digested with BsmBI and ligated with the gRNAs targeting exons 9 and 17 of the *ATP7B* gene. To generate lentivirus, HEK 293T cells were plated at a density of approximately 7.0×10^5 cells in 5 mL of medium in a 6 cm tissue culture plate. A mixture of 1 µg of lentiCRISPRv2 cloned for ATP7B, 750 ng of psPAX2 packaging plasmid (Addgene #12260), and 250 ng of pMD2.G envelope plasmid (Addgene #12259) in 250 µL of serum-free OPTI-MEM (Gibco 01179, CA, USA) was added to 6 µL of TransIT-X2 transfection reagent (#MIR6006, Mirus Bio LLC, Madison, WI, US) and incubated for 15 min at room temperature. Lentiviral particles were prepared 48 h after transfection. To increase the knockout efficiency, two different gRNAs were mixed and used simultaneously. Lentiviral particles were transduced into HepG2 cells that had been plated the day before at a density of approximately 7.0×10^5 cells in a 6 cm tissue culture plate. After 24 h, cells were selected using a fresh medium containing 1 ng/µL of puromycin.

To generate a stable *NAT2* knockdown in SNU387 cells using lentiviral vectors, HEK 293T cells were plated at a density of approximately 7.0×10^5 cells in 5 mL of medium in a 6 cm tissue culture plate one day before transfection. NAT2 short-hairpin RNA (shRNA) (TRCN0000034910) and blank vectors in pLKO.1 were obtained from the human library of the RNAi Consortium (TRC). A mixture of 1 µg of NAT2 shRNA or blank plasmid, 750 ng of psPAX2 packaging plasmid (Addgene #12260), and 250 ng of pMD2.G envelope plasmid (Addgene #12259) in 250 µl serum-free OPTI-MEM (Gibco 01179, CA, USA) was added to 6 µl of TransIT-X2 transfection reagent (#MIR6006, Mirus Bio LLC, Madison, WI, US) and incubated for 30 min at room temperature. Lentiviral particles were

produced via transfection of the above cocktail into HEK 293T cells. Forty-eight hours after transfection, a supernatant containing lentiviral particles was harvested and purified. The lentiviral particles were transduced into SNU387 cells that had been plated the day before at a density of approximately 7.0×10^5 cells in a 6 cm tissue culture plate. After 24 h, cells were selected using a fresh medium containing 1 ng/μl of puromycin (Gibco A11138, CA, USA). After a sufficient selection process (≥4 weeks) with puromycin, stable cells were harvested and used for the subsequent experiments. In addition, knockdown of *ATP7B* was achieved by transfecting ATP7B siRNA (Dharmacon L-019281-00-0005, Lafayette, CO, USA) into SNU387 cells using jetPRIME (Polyplus, 114-15, Illkirch-Graffenstaden, France) and jetPRIME buffer (Polyplus, 712-60, Illkirch-Graffenstaden, France) following the manufacturer's protocol. To confirm the efficiency of gene knockout or knockdown, real-time PCR was performed using sequence-specific primers. Total RNA was extracted from the cells using an AccuPrep® Universal RNA Extraction Kit (Bioneer, Daejeon, Republic of Korea) following the manufacturer's protocol and converted to cDNA using the RNA to cDNA EcoDry™ Premix kit (TaKaRa, Tokyo, Japan). The respective mRNA expression levels of the control and target of interest were compared in each cell, utilizing the $2^{-\Delta\Delta Ct}$ method with four replicates normalized by the housekeeping gene *GAPDH*. The sequence information of gRNA, shRNA, and primers used in this study are listed in **Supplementary Table 10**.

NAT2 and ATP7B overexpression

We examined the gene supplementation effects of NAT2 and ATP7B by introducing pCMV3 vectors containing each cDNA purchased from Sino Biological (#HG11247-CF

and #HG17426-UT, respectively). The mutant plasmids encoding ATP7B-R832, and NAT2*7 (G286E) were generated by a PCR-based site-directed mutagenesis. The flag-tagged NAT2 plasmids were transfected into the HepG2 cells and the effects of NAT2 overexpression were compared with an empty vector transfection. We also exogenously expressed ATP7B WT (K832), ATP7B mutant (R832), NAT2 WT (*4) and NAT2 mutant (*7; G286E) alleles in *ATP7B* KO HepG2 cells to compare their activity. Since these ATP7B expression vectors can be digested by the CRISPR-Cas9 system, we modified the PAM sequences, located in the target exon 9 (TGG to TAG) and 17 (GGG to TGT) to synonymous codon changes, using site-directed mutagenesis (**Supplementary Fig. 2**). Transfection of the plasmids into WT or *ATP7B* KO-HepG2 cells was performed using the TransIT-X2 Dynamic Delivery System (#MIR6006, Mirus Bio LLC, Madison, WI, US), following the manufacturer's protocol.

Fluorescent-activated cell sorting (FACS)

The FACS analyses were used to study the cell death effects of various concentrations of INH (Sigma-Aldrich I3377, St. Louis, USA), copper (II) chloride ($CuCl_2$, Sigma-Aldrich 222011, St. Louis, USA), carbonyl cyanide 4-(trifluoromethoxy)-phenylhydrazone (FCCP, Sigma-Aldrich C2920, St. Louis, USA) on WT or mutant HepG2 cells. The cells were seeded into 12-well microplates at a density of approximately 2.0×10^5 cells/well and incubated overnight to allow attachment. The next day, the cells were treated with each compound for a designated time. The cells were then scraped from the plate with a cell scraper and transferred to a 96-round-bottom plate, washed twice with PBS, and stained with 1) PE-Annexin V (BD Pharmingen, 559763, Sparks, MD, USA) and 7-Amino-

Actinomcycin (7-AAD, BD Pharmingen, 559763, Sparks, MD, USA) to assess the cell death, 2) MitoTracker Green FM (Invitrogen M46750, Carlsbad, CA, USA) and MitoTracker Red CMXRos (Invitrogen M46752, Carlsbad, CA, USA) to measure mitochondrial membrane potential, 3) MitoSOX mitochondrial superoxide indicator (Invitrogen M36008, Carlsbad, CA, USA) to measure mitochondrial superoxide, and 4) BODIPY 581/591 C11 (Lipid peroxidation sensor, Invitrogen D3861, Carlsbad, CA, USA) to measure lipid peroxidation. The cells were incubated for 30 min in a CO_2 chamber (37 °C, 5 % CO_2). After staining, the cells were washed twice with 100 μL PBS with 2 % FBS buffer, resuspended, and analyzed using a flow cytometer (FACS Celesta, BD Biosciences, San Jose, CA, USA).

Western blotting

The WT or mutant HepG2 cells were seeded into 6-well microplates at a density of approximately 1.0×10^6 cells/well and incubated overnight to enable attachment. The next day, the cells were treated with designated concentrations of INH and $CuCl_2$. The cells were lysed with RIPA buffer containing protease and phosphatase inhibitors. Lysate sample concentrations were calculated using a Pierce BCA Protein Assay Kit (Thermo Fisher Scientific, Waltham, MA, USA). Proteins (20 μg) were separated by 4 ~ 12 % Nu-PAGE and transferred to nitrocellulose (NC) membranes (Invitrogen). Blots were blocked with 3 % BSA TBST for 1 h at room temperature, treated with primary antibodies, and incubated overnight at 4 °C. Blots were washed and incubated with HRP-conjugated secondary antibodies for 1 h at room temperature and washed. Bands were visualized using WesternBright ECL western blotting detection reagent (Advansta, Menlo Park, CA,

USA) and FUSION Solo S (Vilber Lourmat, Marne-la-Vallee, France). Purified recombinant HMGB1 protein (rHMGB1) used as positive control was kindly provided by Dr. Jeon-Soo Shin[1]. In order to form oligomeric HMGB1, rHMGB1 and cell lysates were incubated with 100uM CuCl2 and 100uM H2O2 for 2 hours at 37◦C and finally analyzed under non-reducing conditions. The antibodies utilized are specified in **Supplementary Table 11**.

DNA fragmentation assay

The detection of DNA fragmentation was performed using the Apoptotic DNA Ladder Kit (Roche, 11 835 246 001, Germany) as per the manufacturer's procedures. In brief, cells treated with INH and $CuCl_2$ were resuspended in 200ul PBS, added binding/lysis buffer, and mixed immediately. Lysates were incubated for 10 min at room temperature. Cells were then treated with 100 μl isopropanol and transferred to a filter tube and a collection tube. Samples were centrifuged for 1 min at 8,000 rpm and the flow-through was discarded. Samples were added 500 μl of washing buffer to the filter tube and collection tube and centrifuged for 1 min at 8,000 rpm twice. DNA was eluted using an elution buffer, and 2 μg of DNA was loaded onto a 1 % agarose-DNA gel. Bands were visualized using the G:Box System (Syngene, Cambridge, UK).

Viability measurements

The HepG2 or SNU387 cells were seeded into 96-well microplates at a density of approximately 1.0×10^4 cells/well and incubated overnight to enable attachment. The next day, the cells were treated with various concentrations of INH (Sigma-Aldrich I3377, St.

Louis, USA), copper (II) chloride ($CuCl_2$, Sigma-Aldrich 222011, St. Louis, USA), or combinations thereof, of 1 μM and 300 mM on a logarithmic scale. For each condition, more than five replicates were conducted. Cell viability was measured using the WST-8 assay (Quanti-MAX WST-8, Biomax, Seoul, Korea) following the manufacturer's protocol. To summarize, after the incubation period, cells were washed with phosphate-buffered saline (PBS) followed by adding 100 μL of fresh culture medium and 10 μL of assay solution. Absorbance (A) was measured at 450 nm using a 96-well microplate reader (Varioskan Flash 3001; Thermo Fisher Scientific, Inc., MA, USA) after incubation at 37℃ for 4 h. Cell viability (%) was calculated using absorbance data following the equation $(A_{Treated} - A_{Blank})/(A_{Control} - A_{Blank}) \times 100$. The lethal dose (LD50) was calculated from the best-fitted dose-response curve using the least-squares method via the following equation Y = Bottom + (Top-Bottom) / (1+10^((LogLD50 - X)*Hillslope)).

Supplementary References

1. Kwak, M.S. *et al.* Reactive oxygen species induce Cys106-mediated anti-parallel HMGB1 dimerization that protects against DNA damage. *Redox Biol* **40**, 101858 (2021).

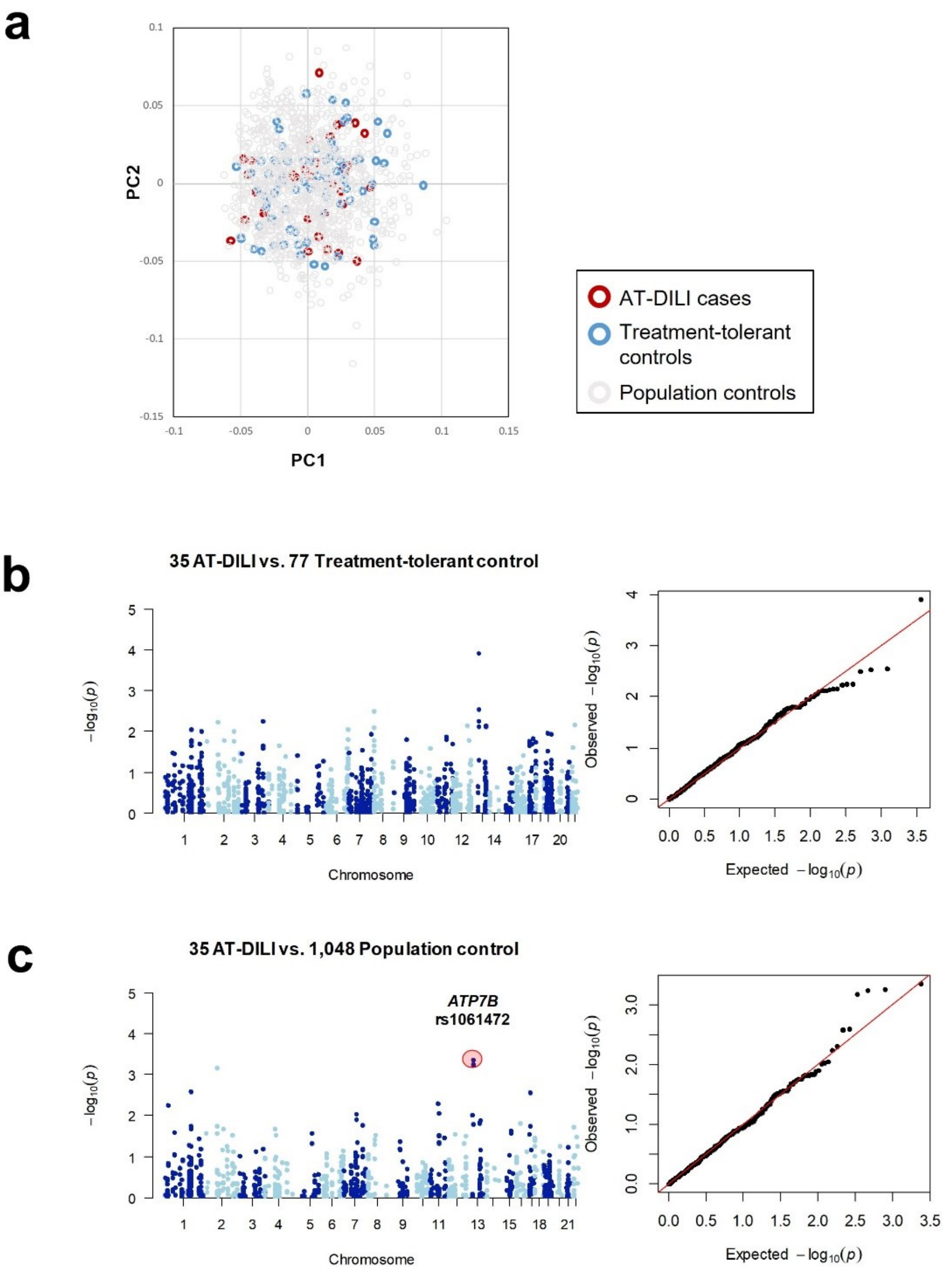

Supplementary Fig. 1. Manhattan plot and quantile-quantile (QQ) plot of Pharmacogenome (PGx)-wide association study (PGxWAS). a Principal Component Analysis (PCA) confirmed the absence of significant bias among the datasets: AT-DILI cases (red circle) and two controls (treatment tolerant control [blue circle], population controls [Korea1K, grey circle]). **b, c** Manhattan plot and quantile-quantile (QQ) plot: AT-DILI case vs. treatment-tolerant control (**b**), and AT-DILI case vs. population control (**c**). The association studies use 1,760 common variants (MAF ≥ 5%) detected in the 380 target pharmacogenes. A significant inflating signal was observed in the QQ plot in comparison with that of the population control, with the strongest association signal in the *ATP7B* K832R variant (rs1061472).

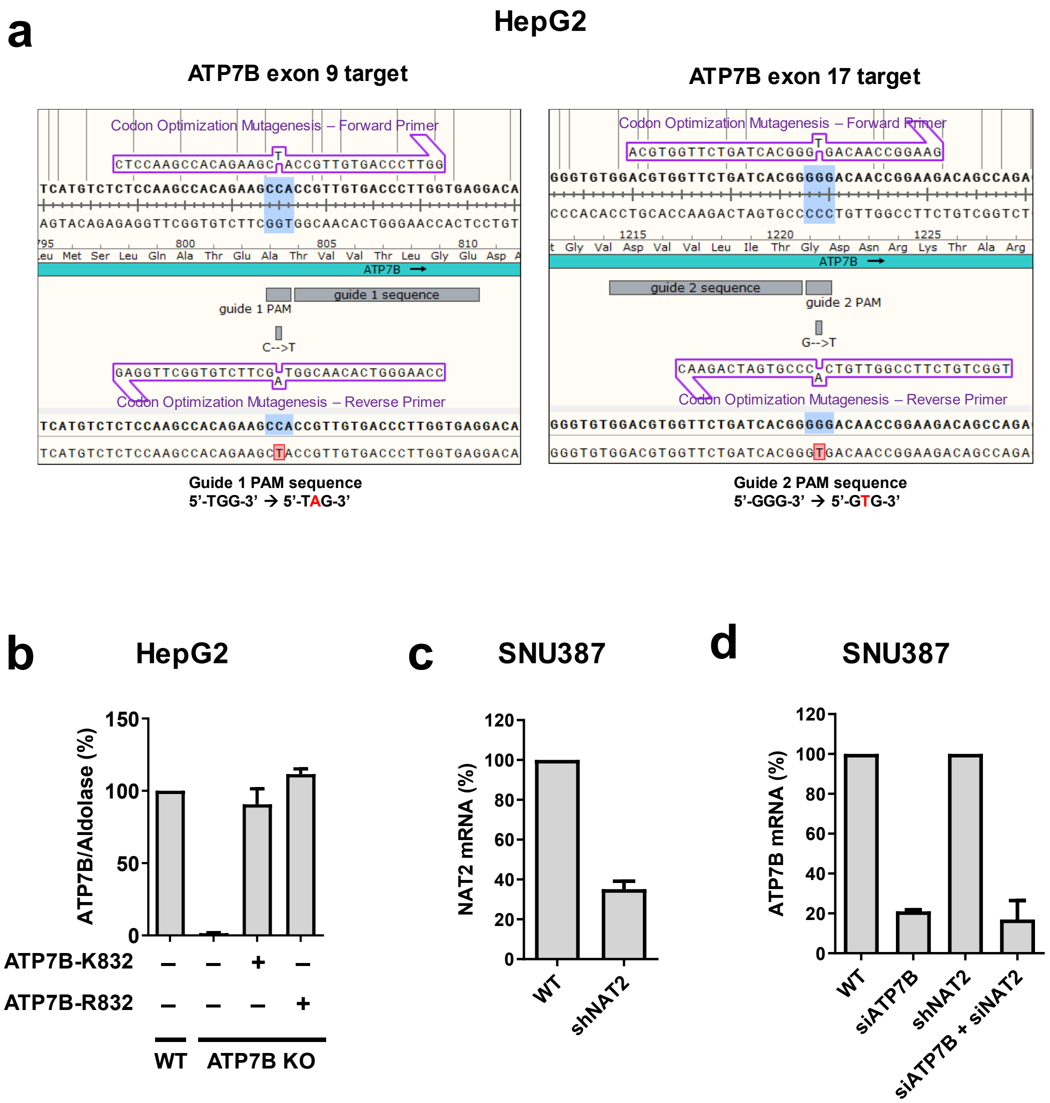

Supplementary Fig. 2. Assessment of gene knockout, knockdown, and supplementation. **a** Generation of ATP7B knock-out (KO) HepG2 cells. The CRISPR-Cas9 system was used to generate *ATP7B* KO cells using the lentiCRISPRv2 vector with the gRNAs targeting exons 9 and 17 of the human *ATP7B* gene. **b** The protein expressions of ATP7B-K832 and ATP7B-R832 in *ATP7B* KO HepG2 cells. An example of Western blotting is shown in Figure 2D and densitometric analyses of multiple experiments are summarized (*n* = 3). For an exogenous expression of wild-type (K832) and mutant (R832) ATP7B proteins in *ATP7B* KO HepG2 cells, the insert ATP7B sequences corresponding to the PAM sequence located in the target exon 9 and 17 regions were modified to a synonymous codon using site-directed mutagenesis (marked in purple, panel A). The similar expression levels of ATP7B-K832 and ATP7B-R832 to that of native ATP7B expression in wild-type (WT) HepG2 cells were achieved by adjusting the dose of each plasmid used for transfection. **c** The gene knockdown efficiency of *NAT2* was analyzed via qPCR in SNU387 cells stably expressing shRNA against human *NAT2* (*n* = 3). **d** The gene knockdown efficiency of *ATP7B* was analyzed via qPCR in wild-type (WT) and shRNA-NAT2 expressing SNU387 cells (*n* = 3). Bar graph data are shown as mean ± SEM.

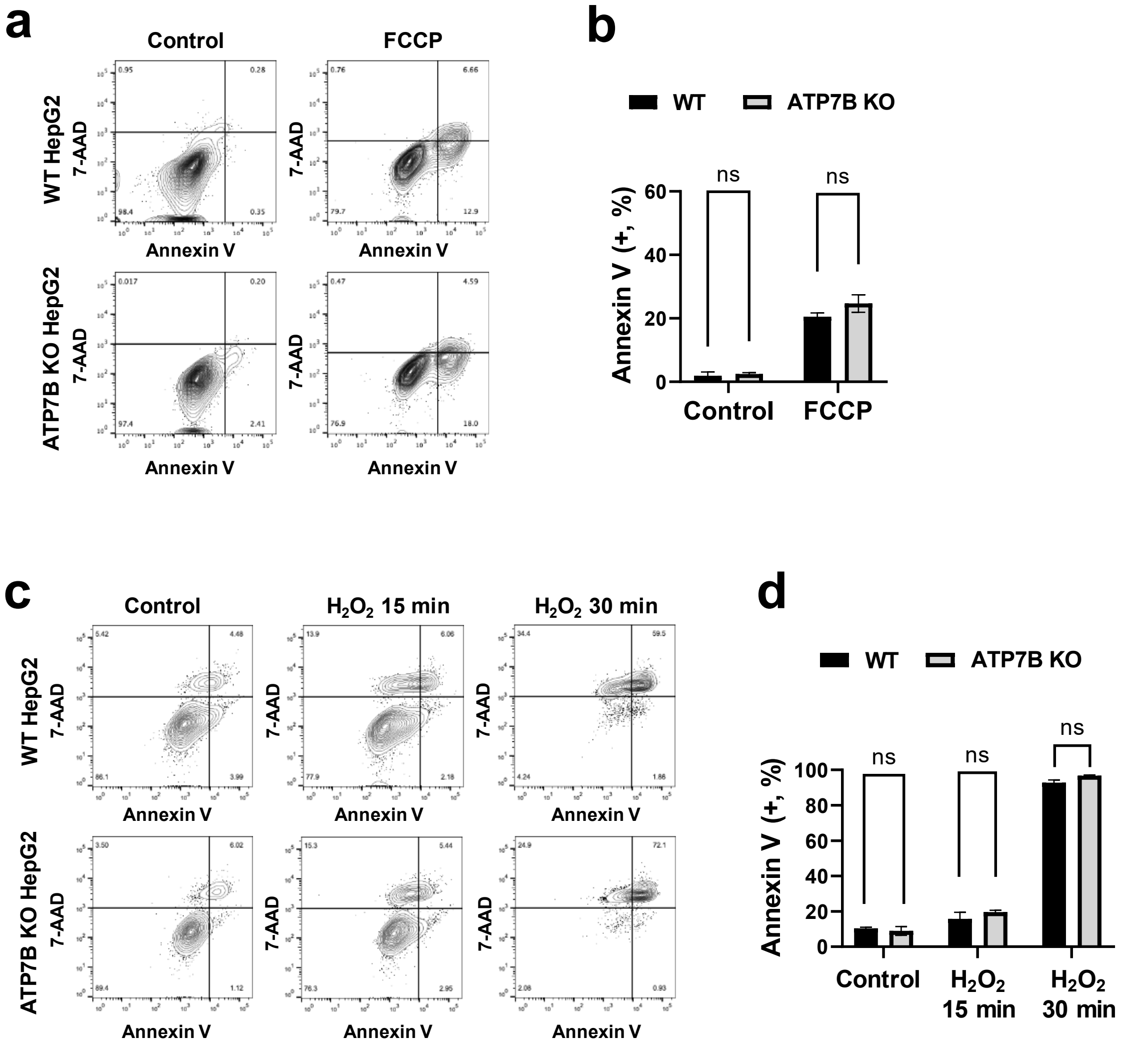

Supplementary Fig. 3. Knockout of ATP7B does not affect the cell injury induced by FCCP and H_2O_2 in HepG2 cells. a, b The cell death patterns were analysed via FACS analyses in HepG2 cells stained with annexin V and 7-AAD. Treating wild-type (WT) and *ATP7B* KO cells with the mitochondrial oxidative phosphorylation uncoupler carbonyl cyanide p-trifluoromethoxy-phenylhydrazone (FCCP, 100 μM) for 24 h resulted in similar levels of annexin V-positive cells in both cell types (n = 3). **c, d** Treating WT and *ATP7B* KO cells with the oxidative stress-inducing agent H_2O_2 (25 mM) for 15 min or 30 min resulted in similar levels of annexin V-positive cells in both cell types (n = 3). Bar graph data are shown as mean ± SEM. ns, not significant.

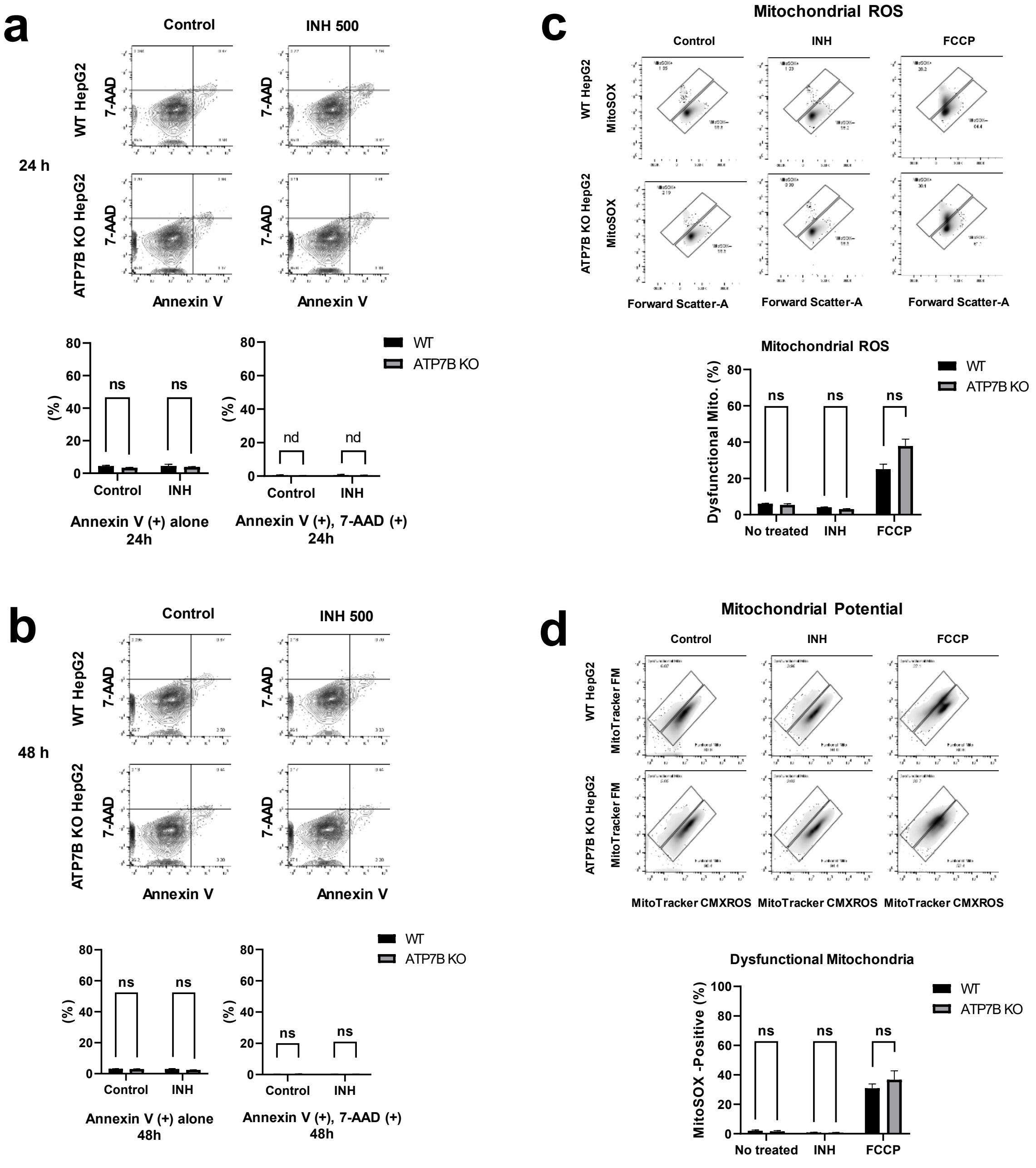

Supplementary Fig. 4. Isoniazid alone does not induce cell death and mitochondrial injury. a, b The cell death patterns were analysed via FACS analyses in HepG2 cells stained with annexin V and 7-AAD. INH alone at concentrations of up to 1,000 μM did not significantly induce cell death in both wild-type (WT) and *ATP7B* KO cells (n = 3-4). **c** Measurements of mitochondrial ROS generation using FACS analyses with MitoSOX as described in Fig. 6a. INH (1000 μM, 24 h) does not induce mitochondrial ROS production (n = 3). **d** Measurement of functional mitochondria (%) using the MitoTracker system as described in Fig. 6c. INH (1000 μM, 24 h) does not increase the number of dysfunctional mitochondria (n = 3). FCCP (25 μM, 24 h) was used as positive controls. Bar graph data are shown as mean ± SEM. ns, not significant by multiple t-tests with the FDR correction.

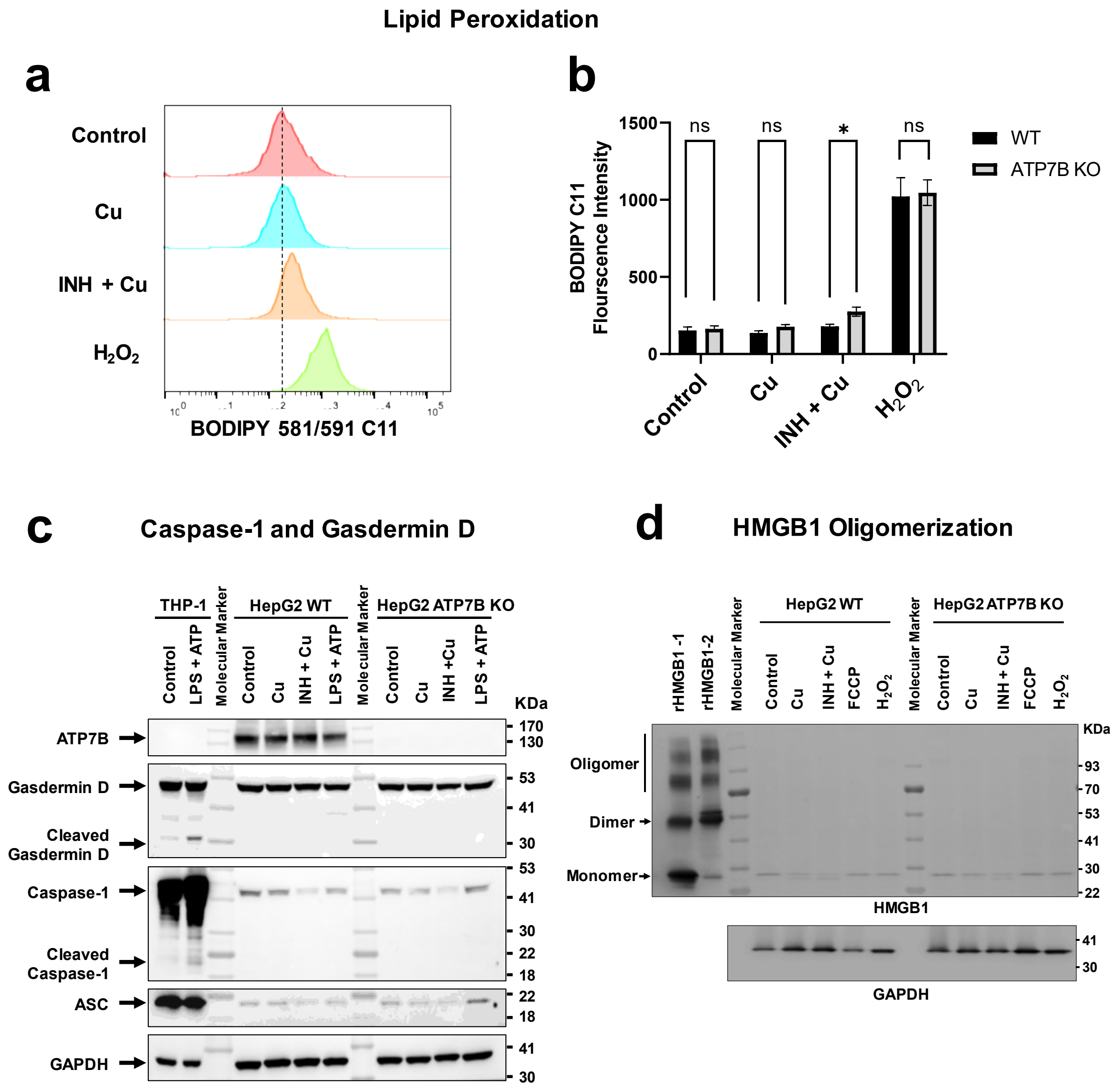

Supplementary Fig. 5. Treatment with INH and Cu does not activate necrotic and pyroptotic cell death signals in HepG2 cells. a, b The FACS analysis of cellular lipid peroxidation using BODIPY 581/591 C11. Treatment with INH and Cu (1,000 and 500 μM, respectively for 24 h) in ATP7B KO cells results in a slight increase in the level of cellular lipid peroxidation, which is only 13 % of that caused by H_2O_2 (25 mM, for 15 min). Bar graph data are shown as mean ± SEM (n = 3). ns, not significant; $*P < 0.05$ by multiple t-tests with the FDR correction. **c** Western-blot analysis of pyroptosis markers, such as cleaved Caspase-1 and cleaved Gasdermin D, shows no significant involvement of pyroptosis pathways in the INH-Cu toxicity. Protein samples from THP-1 cells treated with the lipopolysaccharides (LPS) were used as positive controls. Three independent experiments showed similar results **d** An analysis of HMGB1 oligomerization was conducted via non-reducing SDS-PAGE. No HMGB1 complexes are formed in WT and *ATP7B* KO HepG2 cells following treatment with Cu and INH. The first and second lanes are recombinant HMGB1 protein extracted from *E. Coli.* Three independent experiments produced similar results.

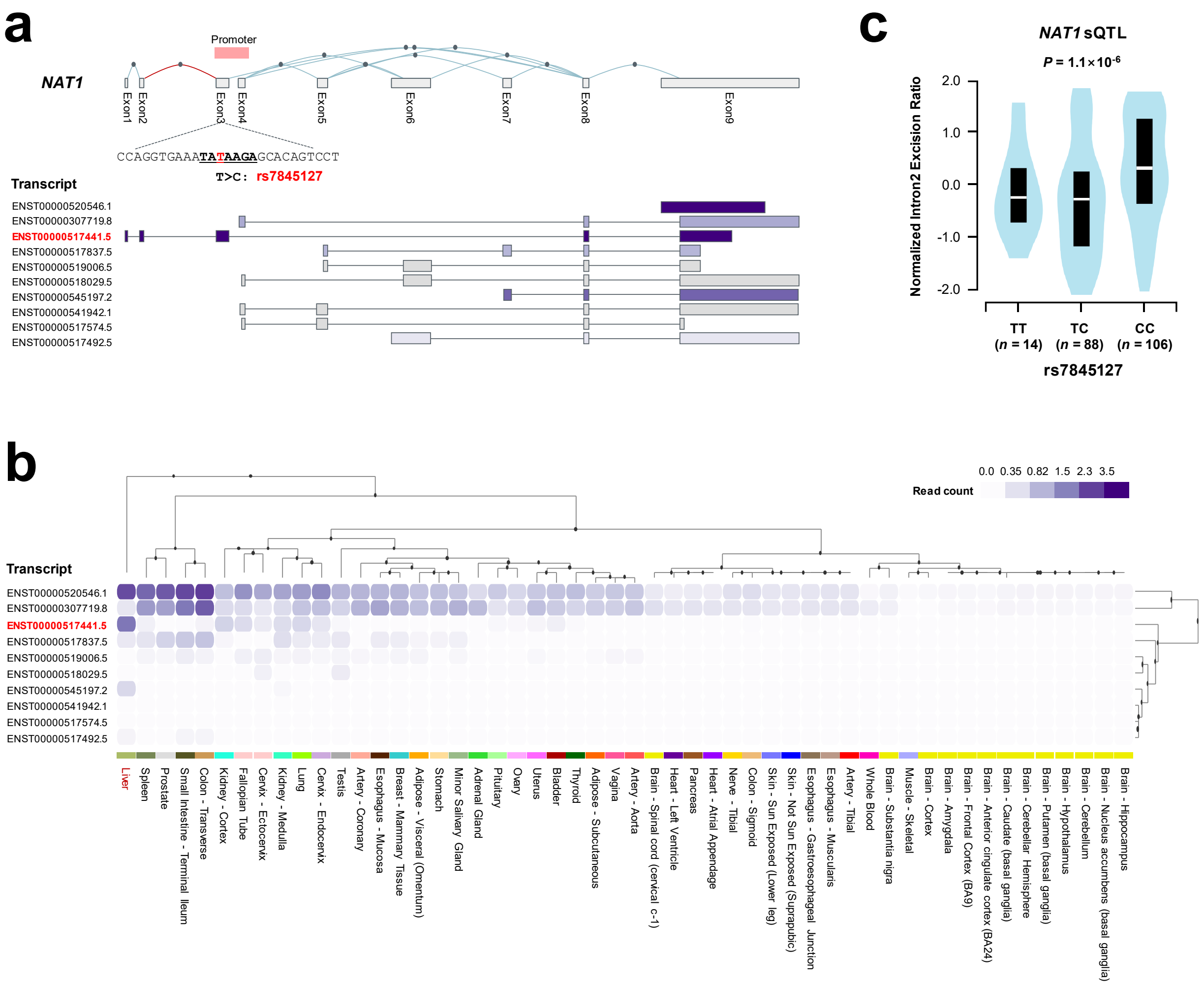

Supplementary Fig. 6. NAT1 variant (rs7845127) as a splicing quantitative trait locus (sQTL) of liver-specific transcript. a The location of NAT1 variant (rs7845127) and the relative expression of NAT1 transcripts in liver. The rs7845127 variant (T>C) is located in exon 3 of the liver specific-transcript (ENST00000517441.5) and promoter region with TATA box sequence (TATAAGA). **b** Relative expression data of NAT1 transcripts across 54 different tissues obtained from the GTEx portal (https://gtexportal.org/) indicate that ENST00000517441.5 is highly expressed in the liver. **c** The rs7845127 variant is a significant splicing quantitative locus (sQTL) of liver-specific transcript (ENST00000517441.5; P = 1.1×10^{-4}) increasing the isoform expression in homozygote alleles.

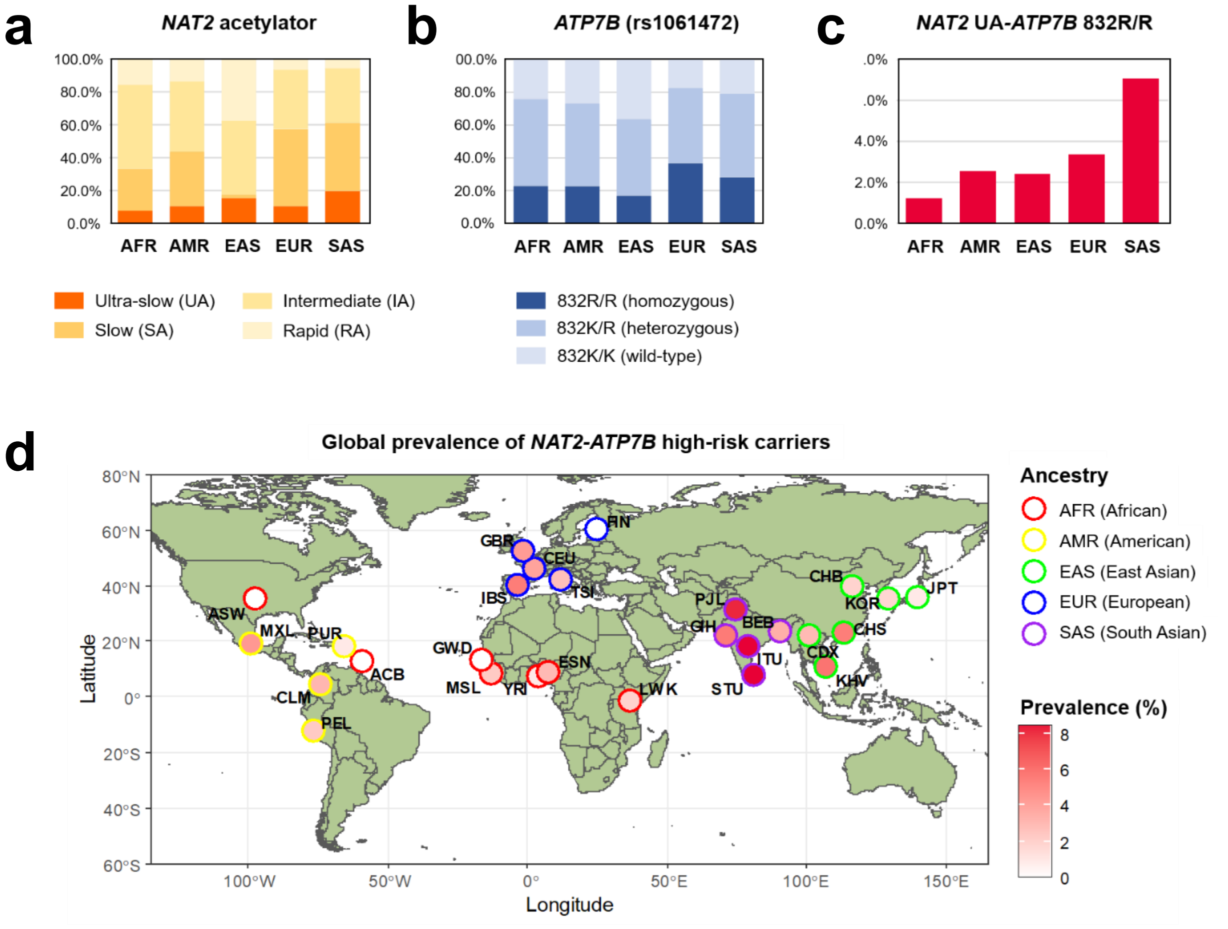

Supplementary Fig. 7. The global prevalence of *NAT2* and *ATP7B* risk alleles. a-c The global frequencies of *NAT2* and *ATP7B* risk alleles were investigated using the Korea1K (n = 1,048) and 1000 Genome (n = 2,535) databases. (**a**) *NAT2* acetylator status, (**b**) *ATP7B* K832R (rs1061472) genotype, and (**c**) the proportions of *NAT2*-ATP7B high-risk genotypes in individuals. African (AFR, 1.2%) and South Asian (SAS, 7.1%) ancestries have, respectively, the lowest and the highest prevalence of these high-risk genotypes in the 1000 Genome databases. **d** Among the 27 different populations, three AFR populations (ASW, ACB, and GWD) and one EUR population (FIN) had no individuals with this high-risk genotype, whereas the three highest populations having frequencies of more than 8% were all observed in the Indian subcontinent (SAS; STU, ITU, and PJL).

Supplementary Table 1. Anti-tuberculosis drug-induced liver injury cases included in this study (*n* = 72)

Patient	Cohort	TB diagnosis	Medication	Sex	Age	DILI type	RUCAM	*NAT2* genotype	*ATP7B* genotype
**TB001**	Discovery	Active TB	HREZ	M	45	Hepatocellular	Probable	*6A/*7B	832K/R
**TB002**	Discovery	Active TB	HREZ	F	38	Hepatocellular	Probable	*4/*4	832K/R
**TB003**	Discovery	Active TB	HREZ	F	33	Mixed	Probable	*4/*7B	832K/R
**TB004**	Discovery	Active TB	HREZ	M	49	Hepatocellular	Probable	*6A/*6A	832R/R
**TB005**	Discovery	Active TB	HREZ	M	43	Hepatocellular	Probable	*4/*6A	832R/R
**TB006**	Discovery	Active TB	HREZ	M	23	Hepatocellular	Probable	*6A/*6A	832R/R
**TB007**	Discovery	Active TB	HREZ	F	35	Hepatocellular	Probable	*4/*4	832K/K
**TB008**	Discovery	Active TB	HREZ	F	24	Hepatocellular	Probable	*6A/*6A	832R/R
**TB009**	Discovery	Active TB	HREZ	M	62	Hepatocellular	Probable	*4/*4	832R/R
**TB010**	Discovery	Active TB	HREZ	F	42	Hepatocellular	Highly Probable	*4/*4	832K/R
**TB011**	Discovery	Active TB	HREZ	M	51	Hepatocellular	Probable	*6A/*7B	832K/K
**TB012**	Discovery	Active TB	HREZ	F	37	Hepatocellular	Probable	*4/*4	832K/R
**TB013**	Discovery	Active TB	HREZ	F	22	Hepatocellular	Highly Probable	*4/*4	832K/R
**TB014**	Discovery	Active TB	HREZ	F	60	Hepatocellular	Probable	*4/*6A	832K/R
**TB015**	Discovery	Active TB	HREZ	M	18	Mixed	Probable	*4/*4	832K/R
**TB016**	Discovery	Active TB	HREZ	M	73	Hepatocellular	Probable	*4/*4	832K/R
**TB017**	Discovery	Active TB	HREZ	F	44	Hepatocellular	Highly Probable	*6A/*6A	832R/R
**TB018**	Discovery	Active TB	HREZ	F	76	Hepatocellular	Probable	*6A/*7B	832K/R
**TB019**	Discovery	Active TB	HREZ	M	46	Hepatocellular	Highly Probable	*4/*4	832K/K
**TB020**	Discovery	Active TB	HREZ	F	46	Hepatocellular	Probable	*4/*4	832R/R
**TB022**	Discovery	Active TB	HREZ	M	76	Hepatocellular	Probable	*4/*4	832K/R
**TB023**	Discovery	Active TB	HREZ	F	69	Hepatocellular	Probable	*4/*6A	832K/K
**TB028**	Discovery	Active TB	HREZ	M	75	Hepatocellular	Probable	*4/*5B	832K/K
**TB036**	Discovery	Active TB	HREZ	F	32	Hepatocellular	Probable	*7B/*7B	832R/R

**TB038**	Discovery	Active TB	HREZ	F	38	Hepatocellular	Probable	*6A/*6A	832R/R
**TB046**	Discovery	Active TB	HREZ	M	33	Hepatocellular	Probable	*4/*4	832K/R
**TB058**	Discovery	Active TB	HREZ	M	84	Hepatocellular	Probable	*6A/*7B	832K/R
**TB068**	Discovery	Active TB	HREZ	F	21	Hepatocellular	Probable	*4/*6A	832K/R
**TB081**	Discovery	Active TB	HREZ	M	48	Hepatocellular	Probable	*4/*7B	832R/R
**TB099**	Discovery	Active TB	HREZ	F	58	Hepatocellular	Probable	*4/*4	832K/R
**TB108**	Discovery	Active TB	HREZ	F	49	Hepatocellular	Highly Probable	*4/*4	832K/R
**TB109**	Discovery	Active TB	HREZ	F	41	Hepatocellular	Probable	*4/*6A	832K/R
**TB110**	Discovery	Active TB	HREZ	M	53	Hepatocellular	Probable	*6A/*6A	832K/R
**TB111**	Discovery	Active TB	HREZ	M	32	Hepatocellular	Highly Probable	*4/*4	832R/R
**TB112**	Discovery	Active TB	HREZ	F	61	Hepatocellular	Probable	*4/*7B	832K/R
**TB114**	Replication	Active TB	HREZ	M	45	Hepatocellular	Probable	*4/*4	832K/R
**TB115**	Replication	Active TB	HREZ	F	40	Hepatocellular	Probable	*4/*4	832R/R
**TB116**	Replication	Active TB	HREZ	F	63	Cholestatic	Probable	*4/*6A	832K/R
**TB117**	Replication	Active TB	HREZ	M	54	Hepatocellular	Probable	*4/*4	832K/K
**TB118**	Replication	Active TB	HREZ	M	67	Mixed	Probable	*4/*6A	832K/R
**TB119**	Replication	Active TB	HREZ	F	38	Hepatocellular	Probable	*6A/*7B	832R/R
**TB120**	Replication	Active TB	HREZ	M	58	Mixed	Probable	*4/*4	832K/K
**TB121**	Replication	Active TB	HREZ	M	74	Cholestatic	Probable	*4/*4	832K/K
**TB137**	Replication	Active TB	HREZ	F	24	Hepatocellular	Probable	*6A/*7B	832R/R
**TB153**	Replication	Active TB	HREZ	M	80	Hepatocellular	Probable	*4/*4	832K/R
**TB166**	Replication	Active TB	HREZ	F	50	Hepatocellular	Probable	*6A/*7B	832K/R
**TB184**	Replication	Active TB	HREZ	F	34	Hepatocellular	Highly Probable	*4/*4	832K/R
**TB185**	Replication	Active TB	HREZ	F	21	Mixed	Probable	*4/*7B	832K/R
**TB186**	Replication	Active TB	HREZ	F	24	Hepatocellular	Probable	*4/*7B	832K/K
**TB188**	Replication	Active TB	HREZ	F	39	Hepatocellular	Probable	*4/*4	832K/K
**TB189**	Replication	Active TB	HREZ	M	74	Mixed	Probable	*4/*4	832K/K

**TB190**	Replication	Active TB	HREZ	M	28	Cholestatic	Probable	*4/*6A	832K/K
**TB195**	Replication	Active TB	HREZ	F	50	Hepatocellular	Probable	*4/*6A	832R/R
**TB196**	Replication	Active TB	HREZ	M	44	Hepatocellular	Probable	*6A/*6A	832K/K
**TB197**	Replication	Active TB	HREZ	M	35	Hepatocellular	Highly Probable	*4/*4	832R/R
**TB199**	Replication	Active TB	HREZ	M	28	Hepatocellular	Highly Probable	*6A/*7B	832K/R
**TB200**	Replication	Active TB	HREZ	M	40	Hepatocellular	Probable	*4/*4	832K/R
**TB201**	Replication	Active TB	HREZ	F	33	Hepatocellular	Probable	*6A/*7B	832K/K
**TB203**	Replication	Active TB	HREZ	F	16	Hepatocellular	Highly Probable	*4/*4	832K/R
**TB204**	Replication	Active TB	HREZ	M	24	Hepatocellular	Probable	*6A/*6A	832R/R
**TB210**	Replication	Active TB	HREZ	M	71	Hepatocellular	Probable	*6A/*7B	832K/K
**TB211**	Replication	Active TB	HREZ	M	69	Hepatocellular	Probable	*4/*7B	832R/R
**TB212**	Replication	Active TB	HREZ	F	72	Mixed	Probable	*4/*7B	832K/R
**TB213**	Replication	Active TB	HREZ	F	49	Hepatocellular	Probable	*4/*7B	832K/K
**TB226**	Replication	Latent TB	INH mono	F	52	Hepatocellular	Probable	*4/*4	832K/R
**TB227**	Replication	Latent TB	INH mono	F	28	Hepatocellular	Probable	*4/*4	832K/K
**TB237**	Replication	Latent TB	INH mono	F	46	Hepatocellular	Probable	*6A/*6A	832R/R
**TB239**	Replication	Latent TB	INH mono	F	49	Hepatocellular	Probable	*4/*4	832K/R
**TB254**	Replication	Latent TB	INH mono	M	49	Hepatocellular	Probable	*4/*4	832R/R
**TB256**	Replication	Latent TB	INH mono	F	51	Hepatocellular	Probable	*4/*6A	832K/R
**TB267**	Replication	Latent TB	INH mono	F	41	Hepatocellular	Probable	*4/*6A	832K/K
**TB272**	Replication	Latent TB	INH mono	F	56	Hepatocellular	Probable	*4/*12A	832K/K

HREZ, isoniazid (H) + rifampin (R) + ethambutol (E) + pyrazinamide (Z); RUCAM, Roussel Uclaf Causality Assessment Method.

Supplementary Table 2. The complete list of 380 target genes

Class	*n*	Gene
Phase I metabolize enzyme	73	*ADH1A, ADH1B, ADH1C, ADH4, ADH6, ADH7, ALDH1A1, ALDH2, ALDH3A1, AOX1, CBR1, CBR3, CES1, CES2, CYP11B1, CYP11B2, CYP17A1, CYP19A1, CYP1A1, CYP1A2, CYP1B1, CYP20A1, CYP24A1, CYP26A1, CYP2A13, CYP2A6, CYP2A7, CYP2B6, CYP2C18, CYP2C19, CYP2C8, CYP2C9, CYP2D6, CYP2E1, CYP2F1, CYP2J2, CYP2R1, CYP2S1, CYP39A1, CYP3A4, CYP3A43, CYP3A5, CYP3A7, CYP4B1, CYP4F11, CYP4F12, CYP4F2, CYP4F3, CYP4F8, CYP4Z1, CYP51A1, CYP7A1, CYP7B1, DPYD, EPHX1, EPHX2, FMO1, FMO2, FMO3, FMO4, FMO5, FMO6P, GPX1, GPX3, GPX4, GSR, GSS, NOS1, NOS3, PON1, PON2, PON3, XDH*
Phase II metabolize enzyme	52	*CHST1, CHST10, CHST11, CHST13, CHST2, CHST3, CHST5, CHST7, CHST8, CHST9, GSTA1, GSTA2, GSTA3, GSTA4, GSTA5, GSTK1, GSTM2, GSTM3, GSTM4, GSTM5, GSTO1, GSTO2, GSTP1, GSTT2, GSTZ1, HNMT, NAT1, NAT2, NNMT, PNMT, SULT1A1, SULT1A2, SULT1B1, SULT1C2, SULT1E1, SULT2A1, SULT2B1, SULT4A1, TPMT, UGT1A1, UGT1A10, UGT1A3, UGT1A4, UGT1A5, UGT1A6, UGT1A9, UGT2A1, UGT2B11, UGT2B15, UGT2B4, UGT2B7, UGT8*
ABC Transporter	14	*ABCA1, ABCB1, ABCB11, ABCB4, ABCB7, ABCC1, ABCC2, ABCC3, ABCC4, ABCC5, ABCC6, ABCC8, ABCG1, ABCG2*
SLC Transporter	41	*SLC10A1, SLC10A2, SLC13A1, SLC15A1, SLC15A2, SLC16A1, SLC19A1, SLC22A1, SLC22A11, SLC22A12, SLC22A13, SLC22A14, SLC22A2, SLC22A3, SLC22A4, SLC22A5, SLC22A6, SLC22A7, SLC22A8, SLC25A27, SLC28A1, SLC28A2, SLC28A3, SLC29A1, SLC29A2, SLC47A1, SLC47A2, SLC5A6, SLC6A3, SLC6A4, SLC6A6, SLC7A5, SLC7A7, SLC7A8, SLCO1A2, SLCO1B1, SLCO1B3, SLCO2B1, SLCO3A1, SLCO4A1, SLCO5A1*
Modifier	20	*AHR, ARNT, ARSA, ATP7A, ATP7B, CAT, CDA, CFTR, KCNJ11, MAT1A, MPO, NR1I2, NR1I3, POR, PPARD, PPARG, RXRA, SERPINA7, SOD2, SOD3*

Pharmacodynamics (PD) genes	180	*ABHD5, ACE, ADA, ADORA2A, ADRB1, ADRB2, AKAP9, ALAS1, ALB, ALOX5, ALPK2, ANO10, AOC1, APOA1, APOA2, ARID5B, ASAH1, BACH1, BAX, BCL2, BDNF, BRCA1, BTLA, C1orf167, CACNA1C, CARD8, CASP1, CASP3, CASP8, CASP9, CCL2, CD274, CD276, CD28, CD40, CD40LG, CD80, CD86, CIAO2B, CLCN6, COMT, CPA6, CPS1, CRIP3, CROT, CTLA4, CYBA, DBH, DCK, DDX10, DPP4, DRD1, DRD2, EGFR, EGFR-AS1, ENTPD1, ESR1, F5, FAAH, FAHD2A, FAS, FASLG, FBXW8, FKBP5, FOXP3, G6PD, GCLC, GCLM, GGT1, GLCCI1, GRK4, GRK5, HAVCR2, HIF1A, HIPK2, HMGCR, HMOX1, HMOX2, HTR1A, HTR2A, ICOS, IDO1, IDO2, IFNGR1, IFNGR2, IL10, IL10RA, IL12A, IL12B, IL12RB1, IL12RB2, IL17A, IL17RA, IL18, IL18R1, IL18RAP, IL19, IL1A, IL1B, IL1R1, IL4, IL4R, IL6, IL6R, KCNE3, KCNH2, KCNIP3, KDM1B, KEAP1, KSR2, LAG3, LDLR, LGALS9, MAOA, MAOB, MED12L, MTHFR, NBR2, NFE2L2, NLRP3, NOS2, NQO1, NR3C1, NR3C2, NT5E, NTRK2, ORM2, P2RY1, P2RY12, PDCD1, PDCD1LG2, PEAR1, PGAP3, PLXNA4, POLD3, PROM2, PRSS53, PSD3, PTGIS, PTGS1, PTGS2, RALBP1, RUNDC3B, RYR1, RYR2, SCN1A, SCN2A, SCN5A, SGF29, SPG7, SRXN1, STAT3, SULT1C4, TBXAS1, TGFB1, TGFBR1, THSD7B, TNFAIP3, TNFRSF14, TNFRSF1A, TNFRSF1B, TNFRSF4, TNFRSF9, TNFSF10, TNFSF14, TNFSF4, TNFSF9, TOP1P1, TPSG1, TRIM43, TXNRD1, TXNRD2, TYMS, TYMSOS, USP44, VDR, VKORC1, VTCN1, ZNF423, ZNF804B*

The target genes were initially selected based on results from a previous study and the PharmGKB database (https://www.pharmgkb.org/). In addition, a number of genes, particularly pharmacodynamic (PD) genes, were included in the present gene panel via literature reviews. *ATP7B* was included because of its involvement in the efflux mechanism of platinum-based drugs (https://www.pharmgkb.org/pathway/PA150642262).

Supplementary Table 3. Overview of pharmacogenetic variants detected in the discovery cohort (*n* = 112)

Location	Total	Common (MAF ≥ 5%)	Less frequent (MAF < 5%)	Novel (dbSNP150)
Exonic	1,782	714	1,068	100
Synonymous	845	390	455	33
Missense	875	306	569	61
Nonsense	17	4	13	0
Splicing junction (± 2bp)	16	8	8	1
Frameshift deletion	10	2	8	3
Frameshift insertion	4	1	3	1
In-frame deletion	8	1	7	1
In-frame insertion	4	1	3	0
Startloss	2	1	1	0
Stoploss	1	0	1	0
5'-UTR (untranslated region)	234	88	146	36
3'-UTR (untranslated region)	828	368	460	112
Upstream	304	148	156	52
Downstream	17	9	8	3
Intronic	962	501	461	70
Total	4,127	1,828	2,299	373

Supplementary Table 4. Assessment of reported markers associated with anti-tuberculosis drug-induced liver injury

Gene	Genotype	Discovery cohort (n = 112)				OR [95% CI]	P value
		Case (n = 35)		Control (n = 77)			
NAT2	UA	11	31.4%	3	3.9%	11.02 [2.62-66.66]	**1.4×10^{-4}**
	RA + IA + SA	24	68.6%	74	96.1%		
CYP2E1	*1A/*1A	23	65.7%	52	67.5%	0.92 [0.37-2.38]	> 0.999
	*1A/*5 + *5/*5	12	34.3%	25	32.5%		
GSTM1	null	16	45.7%	34	44.2%	1.06 [0.44-2.56]	> 0.999
	non-null	19	54.3%	43	55.8%		

RA, rapid acetylator; IA, Intermediate acetylator; SA, slow acetylator; UA, ultra-slow acetylator; OR, odds ratio; CI, confidence interval.

Supplementary Table 5. List of variants (MAF > 5%) associated with anti-tuberculosis drug-induced liver injury (AT-DILI) in the discovery cohort (*P* < 0.01)

Gene	Category	rsID (dbSNP150)	Location	MAF	Population control (*n* = 1,048)		Treatment-tolerant control (*n* = 77)	
					OR [95% CI]	*P* value	OR [95% CI]	*P* value
ATP7B	Modifier	rs1061472	exonic	0.451	2.30 [1.42-3.73]	0.000476	2.21 [1.25-3.94]	0.004717
ATP7B	Modifier	rs732774	exonic	0.455	2.27 [1.40-3.68]	0.00065	2.16 [1.21-3.83]	0.005891
ATP7B	Modifier	rs1801249	exonic	0.455	2.26 [1.40-3.67]	0.000681	2.16 [1.21-3.83]	0.005891
ATP7B	Modifier	rs2277448	5'UTR	0.558	2.45 [1.42-4.21]	0.000865	3.21 [1.72-5.97]	0.000051
PROM2	PD	rs72819488	exonic	0.1339	0.17 [0.05-0.55]	0.00099	0.21 [0.06-0.72]	0.006951
NAT1	Phase II	rs7845127	5'UTR	0.576	0.46 [0.28-0.75]	0.001502	0.42 [0.23-0.74]	0.001728
ATP7B	Modifier	rs1801244	exonic	0.4866	2.02 [1.23-3.30]	0.004422	2.32 [1.30-4.14]	0.002174
NAT1	Phase II	rs2410545	5'UTR	0.589	0.51 [0.31-0.81]	0.005185	0.45 [0.26-0.81]	0.004135
SLCO2B1	Transporter	rs2306168	exonic	0.2723	1.90 [1.16-3.11]	0.007971	2.22 [1.20-4.09]	0.01137
ATP7B	Modifier	rs1801243	exonic	0.5	1.79 [1.09-2.94]	0.02195	2.34 [1.30-4.19]	0.002773
SLCO2B1	Transporter	rs1801906	3'UTR	0.2589	1.71 [1.04-2.80]	0.02828	2.25 [1.21-4.20]	0.008724
SLCO2B1	Transporter	rs41298121	3'UTR	0.2589	1.71 [1.04-2.80]	0.02828	2.25 [1.21-4.20]	0.008724
SLCO2B1	Transporter	rs17133818	3'UTR	0.2366	1.67 [1.01-2.76]	0.04212	2.25 [1.19-4.26]	0.009167
SLCO2B1	Transporter	rs3781727	3'UTR	0.237	1.66 [1.00-2.74]	0.04405	2.25 [1.19-4.26]	0.009167

MAF, minor allele frequency; OR, odds ratio; CI, confidence interval.

Supplementary Table 6. Rare variants detected by gene-based association tests (*P* < 0.05)

Gene	Class	*P* value (SKAT)	*P* value (SKAT-O)	*P* value (Burden)	DILI case	dbSNP ID	Variant Type	Amino acid change	Allele frequency	
									gnomAD exome EAS	Korea1K
GSTM2	Phase II	0.0183	0.0190	0.0165	TB004	rs556730458	Nonsense	K32X	0.000326	0
					TB013	rs371911407	Missense	N59S	0.000979	0.0022
ADH1B	Phase I	0.0183	0.0195	0.0169	TB046	rs760179023	Missense	R313C	0.00008	0.0017
					TB110	rs75967634	Missense	R370H	0.000544	0.0016
HMOX2	PD	0.0183	0.0204	0.0177	TB038	rs183656134	Missense	R285Q	0.0019	0
					TB002	rs140613792	Missense	M288V	0.000435	0
SCN1A	PD	0.0190	0.0202	0.0174	TB038	rs748816300	Missense	V971I	0.000109	0
					TB008	rs121918807	Missense	R1575C	0.000654	0.0055
TNFRSF4	PD	0.0190	0.0194	0.0168	TB019	rs776572462	Missense	P202S	0	0
					TB011	rs762071902	Missense	Q267H	0.000774	0.0028

Supplementary Table 7. Distribution of *NAT2* acetylator status

Acetylator status	Genotype	Discovery cohort (*n* = 112)		Validation cohort (*n* = 165)		Total (*n* = 277)		Korea1K (*n* = 1,048)	Korean population (*n* = 1,000)
RA	*4/*4	50	44.6%	79	47.9%	129	46.6%	41.4%	41.9%
IA	*4/*6A	25	22.3%	31	18.8%	56	20.2%	25.6%	26.4%
	*4/*7B	17	15.2%	31	18.8%	48	17.3%	17.1%	15.4%
	*4/*5B	3	2.7%	2	1.2%	5	1.8%	2.5%	2.3%
	*4/*12A	0	0.0%	2	1.2%	2	0.7%	ND	0.2%
	*4/*19	1	0.9%	0	0.0%	1	0.4%	0.2%	0.6%
	*4/*6C	1	0.9%	0	0.0%	1	0.4%	ND	ND
SA	*5B/*6A	0	0.0%	1	0.6%	1	0.4%	0.4%	0.4%
	*5B/*7B	1	0.9%	0	0.0%	1	0.4%	0.6%	0.3%
UA	*6A/*6A	7	6.3%	8	4.8%	15	5.4%	4.4%	3.9%
	*6A/*7B	5	4.5%	11	6.7%	16	5.8%	5.9%	3.6%
	*7B/*7B	2	1.8%	0	0.0%	2	0.7%	2.0%	1.3%

RA, rapid acetylator; IA, Intermediate acetylator; SA, slow acetylator; UA, ultra-slow acetylator; ND, Not detected.

Supplementary Table 8. Co-occurrence of *NAT2* and *ATP7B* risk genotypes in anti-tuberculosis drug-induced liver injury.

Class	*ATP7B* K832R (rs1061472)	Total	Both genes risk allele	*NAT2* risk only	*ATP7B* risk only	No risk allele	*P* value
DILI cases	Recessive (R/R)	72	10	10	10	42	**0.017**
	Dominant (K/R + R/R)		16	4	37	15	0.558
Treatment-tolerant control	Recessive (R/R)	205	1	31	12	161	0.697
	Dominant (K/R + R/R)		9	4	122	70	0.773
Population control (Korea1K)	Recessive (R/R)	1,048	19	110	128	791	0.787
	Dominant (K/R + R/R)		80	49	571	348	>0.999

Supplementary Table 9. Predictive values of *NAT2* and *ATP7B* risk alleles in the entire cohort (*n* = 277).

Classification	*NAT2* risk allele		*NAT2* and *ATP7B* risk allele	
	+	-	+	-
**DILI case**	20	52	10	62
**Treatment-tolerant control**	13	192	1	204
**Statistics**	**Value (%)**		**Value (%)**	
**Sensitivity**	27.7		13.9	
**Specificity***	93.7		99.5	
**Accuracy**	76.5		77.3	
**PPV**	18.7		60.0	
**NPV**	96.1		95.6	

PPV, positive predictive value; NPV, negative predictive value; The prevalence of DILI is assumed to be 5%.

*Specificity is significantly higher in the NAT2 and ATP7B combination (P = 0.002) than the NAT2 alone, while other statistics show no significant differences.

Supplementary Table 10. Sequence information of gRNA, shRNA and primers used in this study

Name	Target (Accession ID)	Class	Sequence (5' → 3')	Amplicon size (bp)
ATP7B gRNA	ATP7B exon9	gRNA	CTCACCAAGGGTCACAACGG	-
	ATP7B exon17	gRNA	ATTGACCCCATCCCCCACCA	-
NAT2 shRNA	TRCN0000034910	shRNA target sequence	GTCTCCAACATCTTCATTTAT	-
ON-TARGET plus Human ATP7B siRNA		siRNA	GAUAUUGAGCGGUUACAAA	-
			GCGUGGCAGUCACCAAAUA	-
			CAAAGCCCUUGUUAAGUUU	-
			AAGAGGCCGUCAUCACUUA	-
GAPDH mRNA	NM_001256799	Forward primer	GTCTCCTCTGACTTCAACAGCG	235
		Reverse primer	ACCACCCTGTTGCTGTAGCCAA	
ATP7B mRNA	NM_001243182	Forward primer	GGCCGTCATCACTTATCAGCC	103
		Reverse primer	GGGAGCCACTTTGCTCTTGA	
NAT2 mRNA	NM_000015	Forward primer	ACCTGGACCAAATCAGGAGAG	116
		Reverse primer	TGTTCGAGGTTCAAGCGTAAAT	
ATP7B-R832	rs1061472	Forward primer	GCGGGGCGATATCGTCAGGGTGGTCCC	
		Reverse primer	GGGACCACCCTGACGATATCGCCCCGC	
NAT2 UA (*7; G286E)	rs1799931	Forward primer	CGTGCCCAAACCTGGTGATGAATCCCTTAC	
		Reverse primer	CCAATAGTAAGGGATTCATCACCAGGTTTG	

Supplementary Table 11. The complete list of antibodies used in this study

Name	Manufacturer	Catalog number
Anti-Caspase-3	Cell Signaling Technology (Danvers, MA, USA)	#14220
Anti-Cleaved Caspase-3	Cell Signaling Technology (Danvers, MA, USA)	#9664
Anti-Caspase-9	Cell Signaling Technology (Danvers, MA, USA)	#9508
Anti-Cleaved Caspase-9	Cell Signaling Technology (Danvers, MA, USA)	#52873
Anti-PARP	Cell Signaling Technology (Danvers, MA, USA)	#5625
Anti-Gasdermin D	Cell Signaling Technology (Danvers, MA, USA)	#69469
Anti-FLAG	Cell Signaling Technology (Danvers, MA, USA)	#14793
Anti-HMGB1	Abcam (Cambridge, USA)	ab18256
Anti-ATP7B	Abcam (Cambridge, USA)	ab131208
Anti-Caspase-1	Abcam (Cambridge, USA)	ab207802
Anti-GAPDH	Santa Cruz Biotechnology (Dallas, TX, USA)	sc-47724
Anti-NAT2	GeneTex (Irvine, CA, USA)	#GTX114051
Anti-Aldolase	GeneTex (Irvine, CA, USA)	#GTX101408
